# The lipid components of high-density lipoproteins (HDL) are essential for the binding and transportation of antimicrobial peptides in human serum

**DOI:** 10.1038/s41598-022-06640-7

**Published:** 2022-02-16

**Authors:** Wen-Hung Tang, Shi-Han Wang, Chiu-Feng Wang, Yun Mou, Min-Guan Lin, Chwan-Deng Hsiao, You-Di Liao

**Affiliations:** 1 Institute of Biomedical Sciences, Academia Sinica, Taipei, 115 Taiwan; 2 Institute of Molecular Biology, Academia Sinica, Taipei, 115 Taiwan

**Keywords:** Microbiology, Molecular biology, Biochemistry

## Abstract

Antimicrobial peptides (AMPs) have been developed for the treatment of bacterial infections, but their applications are limited to topical infections since they are sequestered and inhibited in serum. Here we have discovered that the inhibition of AMPs by human serum was mediated through high-density lipoproteins (HDL) which are known to remove cholesterol from peripheral tissues. The susceptibility of AMPs to HDL varied depending on the degree of hydrophobicity of AMPs and their binding affinities to HDL. The phospholipids, such as phosphatidylcholine, of HDL were essential for AMP-binding. The dynamic binding interactions between AMPs and HDL were mediated through the hydrophobic interactions rather than by ionic strength. Interestingly, some AMPs, such as SMAP29, dissociated from the AMP-HDL complex and translocated to bacteria upon contact, while some AMPs, such as LL37, remained in complex with HDL. These results suggest that HDL binds AMPs and facilitates the translocation of them to the bacteria.

## Introduction

The widespread use of antibiotics in both medicine and agriculture has contributed to the emergence of drug-resistant bacteria ^1,2^. Thus, development of new antimicrobials with unique targets and action mechanism that are different from those of conventional antibiotics is urgently needed. Natural antimicrobial peptides/proteins (AMPs) with the ability to disrupt membrane integrity have been isolated from multiple sources^2–4^. Several AMPs are currently under examination in phase II/III clinical trials for their effectiveness in preventing and/or treating microbial infections, such as Omiganan (Migenix), Pexigana acetate (MacroChem) and Iseganan (Ardea Biosciences)^5–7^. However, their applications are limited primarily to topical infections because in circulation they are inhibited by serum proteins.

In human serum, albumin is the most abundant protein (45 mg/ml), followed by α1-antitrypsin, transferrin, α2-macroglobulin, apolipoprotein B (Apo-B, major component of low density lipoproteins (LDL)), α1-acid glycoprotein and apolipoprotein A-I (Apo-AI, major component of high density lipoproteins (HDL))^8^. They are involved in the transportation of fatty acids, cholesterols, toxins and drugs in the blood. Drugs are effective in mediating their pharmacological activity and/or side effects only while in the free, unbound form. Often, they may be sequestered by serum proteins, preventing them from achieving high levels of exposure in the systemic circulation. Many small molecule drugs are known to be sequestered and transported by albumin in human serum. For example, more than 90% of anticoagulation drug warfarin and antipyretic drug ibuprofen were found to bind to human albumin at drug binding site I and site II, respectively^9–11^. In addition, the majority (90–94%) of the lipopeptide antibiotic, daptomycin, was found to be bound and inhibited by albumin, while only a small portion was bound by lipoproteins, such as LDL and HDL^12^.

In this study, we found that several AMPs were bound and inhibited by HDL in human serum. We also found that some apparently weaker-bound AMPs translocated from the AMP-HDL complex to bacteria upon contacting the bacteria.

## Results

### Inhibition and binding of antimicrobial peptide (AMP) by human serum

**Figure 1:**
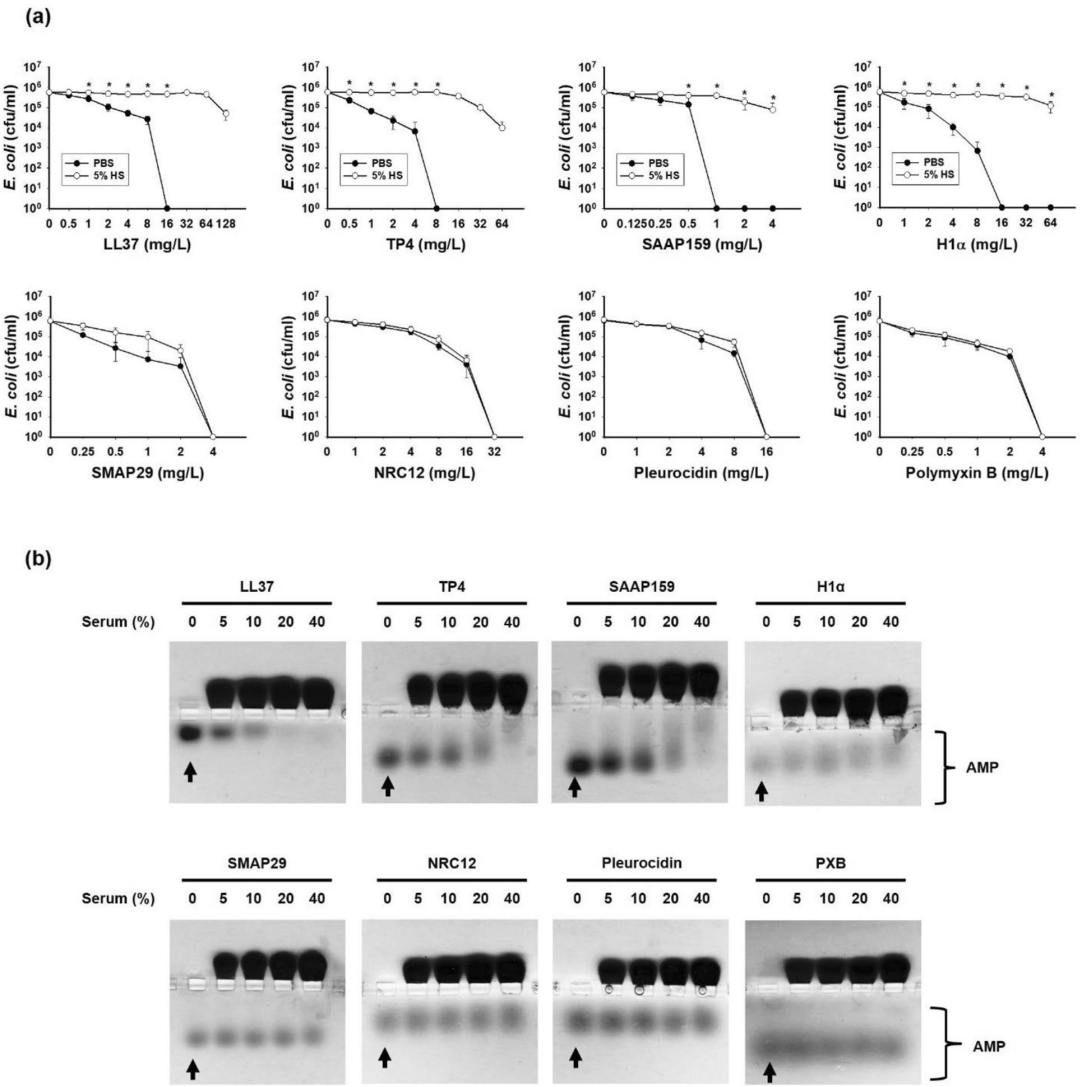
Susceptibility of antimicrobials to human serum inhibition. (**a**) Effect of human serum on the bactericidal activities of antimicrobials against *E. coli* (10^6^ cfu/ml). Values are the mean ± SD (n = 3). Mann–Whitney tests were performed to determine the significance of the difference. * Indicates *P* ≤ 0.05 compared with PBS group. (**b**) The band shift of antimicrobials (4 µg each) in 10 µl diluted human serum analyzed by 8% horizontal native PAGE and Coomassie blue staining. Arrow indicates control (non-shift) AMP. PBS, phosphate-buffered saline; 5% HS, 5% human serum in PBS (v/v).

The antimicrobial activities of AMPs, such as LL37 from human, TP4 from fish tilapia, SAAP159 derived from LL37 and synthetic peptide H1α against *E. coli* were markedly repressed in the presence of 5% human serum (v/v) in comparison with those measured in phosphate buffered saline (PBS) (Fig. 1a top panels)*.* However, these serum-mediated repressions of AMPs’ activities were only occasionally observed with SMAP29 from sheep and not at all observed with NRC12 from American plaice, Pleurocidin from *Pleuronectes amertcanus* or with the peptide-containing antibiotic, Polymyxin B (Fig. 1a bottom panel). To investigate whether these repressions are mediated through the binding of serum components, the mobility-shift of each AMP was analyzed by horizontal native PAGE. Interestingly, the band shift of various AMPs in 5–40% (v/v) serum was positively associated with the susceptibility of AMP to serum inhibition (Fig. 1b). For examples, LL37 was completely shifted in 20–40% serum, while NRC12, Pleurocidin and Polymyxin B (PXB) were not shifted at the serum concentrations tested.

Alternatively, the inhibition of AMP’s bactericidal activity in 5% serum was also determined by cfu counting method and expressed as LC_99_. As shown in Supplementary Table S1, the LC_99_ of LL37 in 5% human serum (> 512 mg/L) was much higher (> 32 fold) than that measured in PBS (16 mg/L). The higher LC_99_ values were also found with other AMPs, such as TP4 (16-fold), RRIKA (16-fold), SAAP159 (16-fold), and H1α (eightfold), but was not found with SMAP29, NRC12 or Pleurocidin (onefold).

### Purification of serum components responsible for the binding and inhibition of AMP

**Figure 2:**
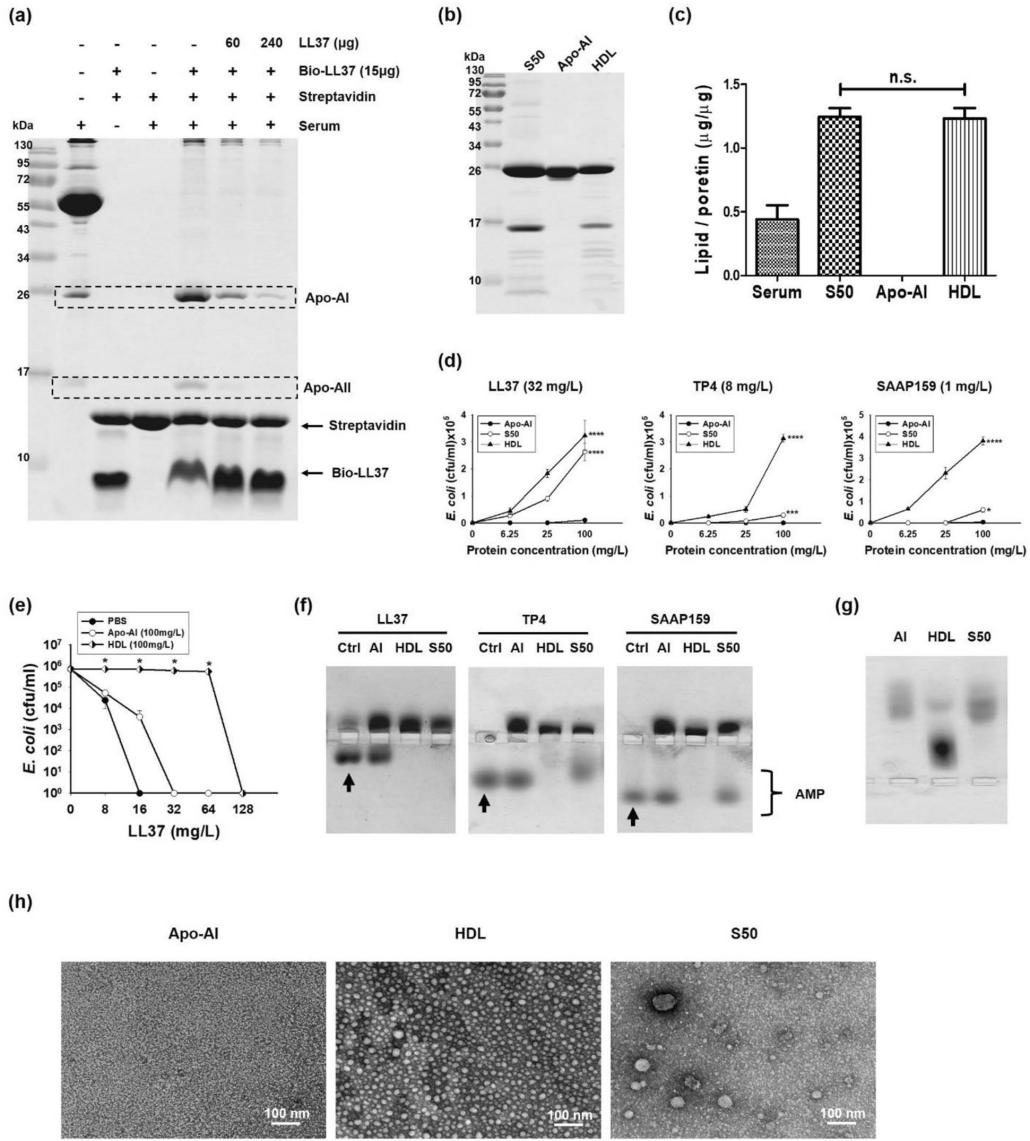
HDL was responsible for AMP-inhibition. (**a**) Specific serum proteins bound to biotinylated LL37. The components of crude human serum were pulled down by immobilized LL37 peptides and analyzed by 15% non-reducing SDS-PAGE and Coomassie blue staining. (**b**) Compositions of purified proteins analyzed by 15% non-reducing SDS-PAGE and Coomassie blue staining. (**c**) Lipid contents of serum, S50, Apo-AI and HDL. Significance is calculated according to One-way ANOVA test. n.s. indicates no significant difference. (**d**) Inhibition of bactericidal activity of AMP against *E. coli* (10^6^ cfu/ml) by various agents. Values are the mean ± SD (n = 3). Kruskal–Wallis tests were performed to determine the significance of the difference in each group. * Indicates *P* ≤ 0.05, *** Indicates *P* ≤ 0.0005, **** Indicates *P* ≤ 0.00005. (**e**) Effects of HDL and Apo-AI on the bactericidal activity of LL37 against *E. coli* (10^6^ cfu/ml). Values are the mean ± SD (n = 3). Mann–Whitney tests were performed to determine the significance of the difference. * Indicates *P* ≤ 0.05 compared with PBS group. (f). Band shift of AMPs (4 µg each) by various agents (10 µg each) analyzed by 8% horizontal native PAGE, pH8.0, and Coomassie blue staining. Arrow indicates control (non-shift) AMP. (**g**) Mobility of various agents (4 µg each) on horizontal 8% native PAGE, pH8.8, and Coomassie blue staining. (**h**) Morphology of purified agents analyzed by TEM.

To determine the serum components responsible for AMP-binding, crude human serum was incubated with biotinylated AMPs followed by Streptavidin-conjugated beads. Two proteins with molecular weight of approximately 26 kDa and 17 kDa, respectively, were pulled down by the immobilized LL37, TP4, SMAP29, H1α and SAAP159, but not by NRC12 or Pleurocidin (Supplementary Fig. S1). The binding of these two proteins to biotinylated LL37 was specifically reduced if excess amounts of exogenously added free form LL37 was preincubated with the serum (Fig. 2a). These two proteins were identified to be apolipoprotein A-I and A-II (abbreviated as Apo-AI and Apo-AII), respectively, after LC/MS/MS analysis (Supplementary Table S2). Apo-AII existed in dimeric form under non-reducing environments. The binding of these two proteins to biotinylated LL37 was resistant to NaCl even up to 1M, but susceptible to 20% trifluoroethanol (TFE, v/v) and 0.3% anionic surfactant sarkosyl (w/v) (Supplementary Fig. S2). Therefore, these two proteins as well as other possible associated components were purified by incubating the serum with immobilized LL37 and eluting with 50% TFE, then vacuum dried and solubilized in PC buffer (20 mM HEPES, pH7.4, 50 mM NaCl) containing 2% TFE and named S50. The major protein components as well as total lipid contents of S50 were found to be similar to those of commercially available human HDL (Fig. 2b,c). The bactericidal activities of LL37, TP4 and SAAP159 were dramatically repressed by HDL in a dose-dependent manner. However, only the bactericidal activities of LL37 were repressed by S50, but those of TP4 and SAAP159 were not significantly repressed. Furthermore, the bactericidal activities of all of the tested AMPs were not repressed by commercially available Apo-AI alone in which no lipids were observed (Fig. 2d). The bactericidal activity of LL37 was also apparently repressed by HDL, but not by Apo-AI (100 mg/L) in a LL37-dependent manner (Fig. 2e). Similar to the bactericidal activity findings, LL37 (4 µg) was bound by both HDL and S50 (10 µg each) as analyzed by band-shift assay, while TP4 and SAAP159 were bound by HDL only, and not by S50. Again, all three AMPs were not bound by free Apo-AI alone at the same concentration (Fig. 2f). To investigate the possible reason for the reduced inhibitory effects of S50 on AMP, the mobility of S50 on native PAGE, pH8.8, was examined. The result shows that S50 exhibited similar mobility to Apo-AI, but faster than HDL (Fig. 2g). It indicates that HDL is dissociated into individual components after TFE elution although the protein compositions and total lipid contents of S50 are similar to those of HDL. By TEM examination it was found that the HDL existed in small vesicles with diameters ranging from 10 to 20 nm, while most of S50 existed in minute particles as did Apo-AI (Fig. 2h). Some of the S50 components existed in large vesicles that are likely lipid droplets, whose sizes ranged from 40 to 50 nm.

### Lipids of HDL are responsible for AMP-binding

**Figure 3:**
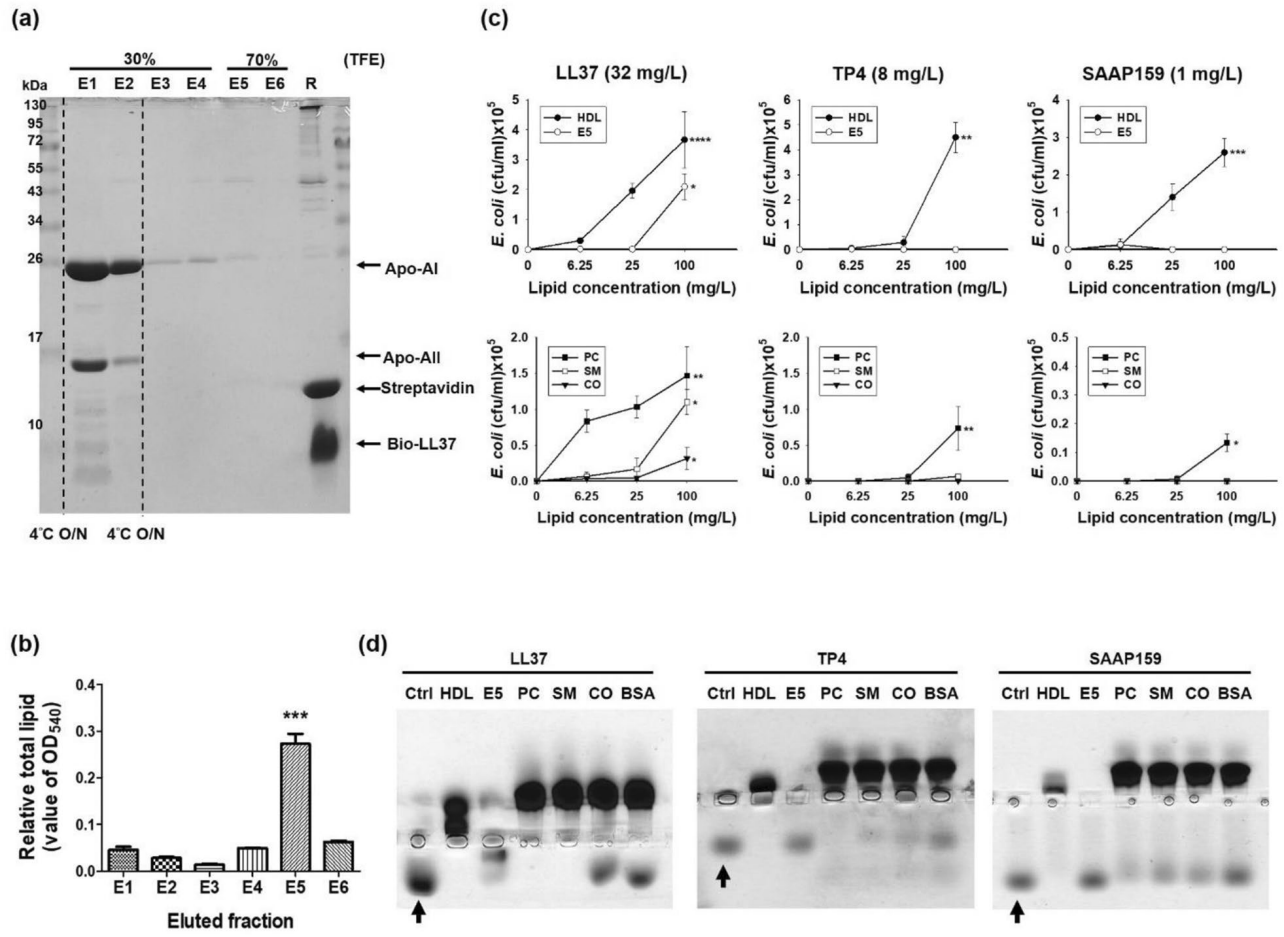
Roles of lipids in the HDL for AMP-binding. (**a**) Purification of human serum components responsible for AMP-binding. The human serum components were pulled down by immobilized LL37, stepwise eluted by 30% and 70% TFE after overnight incubation at 4 °C, and analyzed by 15% non-reducing SDS-PAGE. (**b**) Lipid contents of the eluates (E1–E6). Significance is calculated according to One-way ANOVA test. *** indicates *P* ≤ 0.0005 which E5 compared with each other groups. (**c**) Effect of HDL, eluate E5 (top panel) and various lipids (bottom panel) on the antimicrobial activities of AMPs against *E. coli* (10^6^ cfu/ml). Values are the mean ± SD (n = 3). Kruskal–Wallis tests were performed to determine the significance of the difference in each group. * Indicates *P* ≤ 0.05, ** Indicates *P* ≤ 0.005, *** Indicates *P* ≤ 0.0005, **** Indicates *P* ≤ 0.00005. (**d**) Band shift of AMPs by 10 µg HDL, E5 and various lipids (4 µg each, dissolved in 20 µg/10 µl BSA) and analyzed by 8% horizontal native PAGE and Coomassie blue staining. Arrow indicates control (non-shift) AMP. PC: phosphatidylcholine; SM: sphingomyelin; CO: cholesteryl oleate; BSA: bovine serum albumin.

The proteins and lipids of serum immobilized on biotinylated LL37 were stepwise-eluted with 30% and 70% TFE as indicated after overnight incubation at 4 °C. Most LL37-bound proteins, such as the Apo-AI and Apo-AII, were eluted by 30% TFE and collected in the E1 and E2 fractions, while most lipids were eluted by 70% TFE and collected in the E5 fraction (Fig. 3a,b). The bactericidal activity of LL37 was repressed by the lipid-rich E5 fraction at the indicated concentration (100 mg/L), while those of TP4 and SAAP159 were not repressed (Fig. 3c top panel). To investigate which lipid component of HDL is responsible for AMP-binding, the major lipids of HDL, phosphatidylcholine (PC), sphingomyelin (SM) and cholesteryl oleate (CO) were each dissolved in bovine serum albumin (BSA)-containing buffer and assayed for their ability to inhibit the bactericidal activities of LL37, TP4 and SAAP159 (Fig. 3c bottom panel). The results show that PC, which belongs to the phospholipid class (37–49% in mol% of total HDL lipids), was the most effective. SM, which belongs to the sphingolipid class (5–7%) was less effective, while CO, which belongs to the neutral lipid class (47–54%), was the least effective. Similar results were found with these lipids’ binding abilities to LL37, TP4 and SAAP159 (4 µg each), where PC was also the most effective, followed by SM (Fig. 3d). Consistent with our findings that NRC12 and Pleurocidin were resistant to inhibition and band shift by crude human serum (Fig. 1), we further demonstrated that both the bactericidal activities and band shifts of NRC12 and Pleurocidin were resistant to purified S50, E5, Apo-AI, PC, SM, CO and even to HDL (Supplementary Fig. S3). It is of note that SMAP29 was slightly shifted by HDL, but not by other purified proteins or lipids (Supplementary Fig. S3b,c).

### Hydrophobicity of AMP is critical for HDL-binding

To investigate the differential susceptibilities of AMPs to the inhibition and binding by HDL or lipid components, the hydrophobicity of LL37, TP4 and SAAP159 were determined by assessing their binding to 8-anilino-1-naphthalenesulfonic acid (ANS). ANS exhibits a blue shift in emission wavelength from 520 to 470 nm upon binding to a hydrophobic substance. Upon incubating with increasing concentrations of ANS, we found that LL37 exhibited a clear blue shift in 10% trifluoroethanol (TFE), while TP4 and SAAP159 did not (Supplementary Fig. S4).

**Figure 4:**
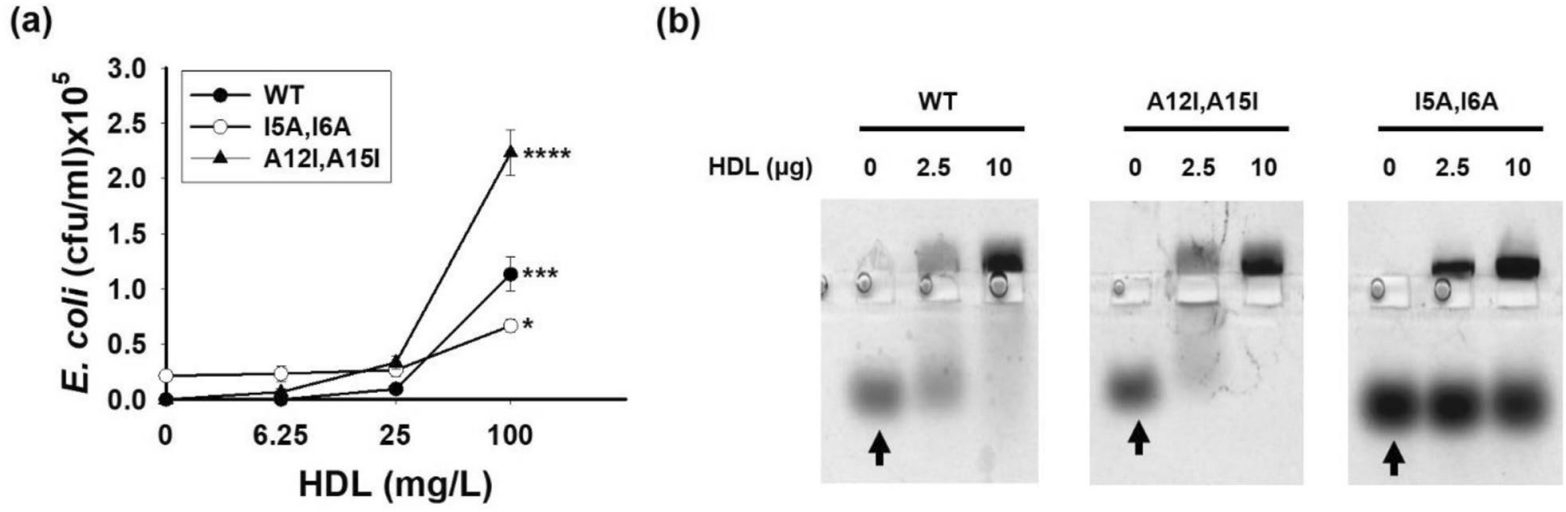
Hydrophobic residues of TP4 were responsible for its binding to HDL (**a**) Effect of HDL on the antimicrobial activities of TP4-WT, TP4-I5A,I6A and TP4-A12I,A15I (40 mg/L each) to *E. coli* (10^7^ cfu/ml). Values are the mean ± SD (n = 3). Kruskal–Wallis tests were performed to determine the significance of the difference in each group. * Indicates *P* ≤ 0.05, *** Indicates *P* ≤ 0.0005, **** Indicates *P* ≤ 0.00005. (**b**) Band shift of TP4 variants (4 µg) by HDL and analyzed by 8% horizontal native PAGE and Coomassie blue staining. Arrow indicates control (non-shift) AMP.

Next, we altered the hydrophobicity of TP4 which shows moderate susceptibility to HDL, and analyzed the consequences. We found that the introduction of higher hydrophobicity amino acid substitutions (TP4-A12I, A15I) increased the AMP’s band shift and susceptibility to the inhibition by HDL. Conversely, a reduction in hydrophobicity via substituting with lower hydrophobicity amino acids (TP4-I5A, I6A) decreased both activities (Fig. 4). These results suggest that the hydrophobic residues as well as the hydrophobicity of AMP is essential for HDL binding.

### Disruption of the HDL complex by competitive addition of AMPs

**Figure 5:**
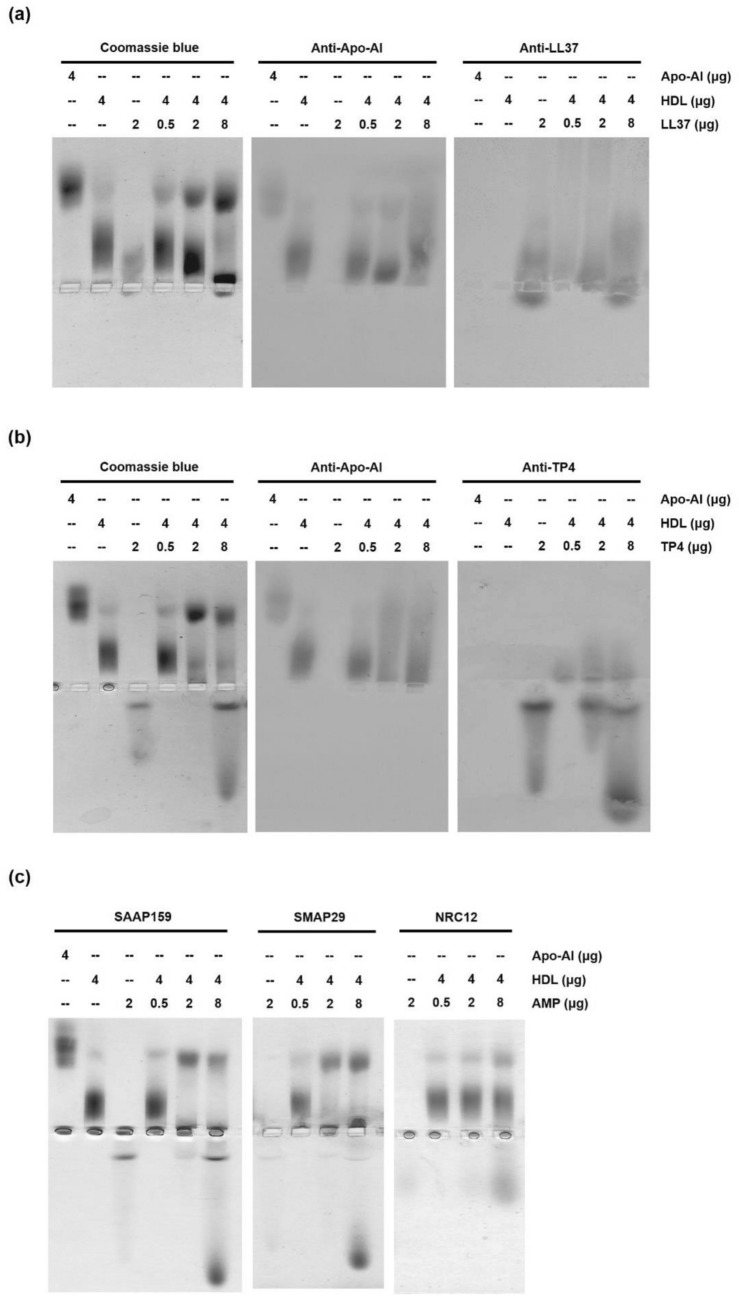
Destructive effect of AMP on HDL (**a, b**) Band shift of HDL cause by TP4 (**a**) or LL37 (**b**) analyzed by 8% horizontal native PAGE, pH8.8, followed by Coomassie blue staining (left) and Western blotting using anti-Apo-AI (middle) and anti-AMP (right) antibodies. (**c**) Band shift of HDL cause by SAAP159 (left), SMAP29 (middle) and NRC12 (right) analyzed by 8% horizontal native PAGE, pH8.8, and Coomassie blue staining.

To determine the consequences of HDL in contact with AMP, we performed the experiments by incubating varying concentrations of AMPs with HDL (0.4 mg/ml) within the physiological concentrations (0.35–0.85 mg/ml). These AMPs, such as LL37 and TP4, bound to HDL at a low AMP:HDL ratio of 1:8 (0.05/0.4 mg/ml, protein base). However, Apo-AI protein dissociated from the HDL complex if excess amounts of AMP were employed at higher AMP:HDL ratios of 1:2 and 2:1, respectively (0.2/0.4 mg/ml, 0.8/0.4 mg/ml, protein base), as analyzed by horizontal native PAGE, pH 8.8, followed by Western blotting using anti-Apo-AI, anti-LL37 and anti-TP4 antibodies (Fig. 5a,b). It is of note that some Apo-AI proteins may still associate with the core of HDL or HDL-AMP complex after high AMP treatment. In addition, SMAP29 and SAAP159 also disrupted the HDL complex and released Apo-AI at higher AMP:HDL ratios of 1:2 and 2:1, respectively (protein base), while NRC12 minimally affected HDL (Fig. 5c).

### Translocation of AMP from AMP-HDL complex to bacteria

**Figure 6:**
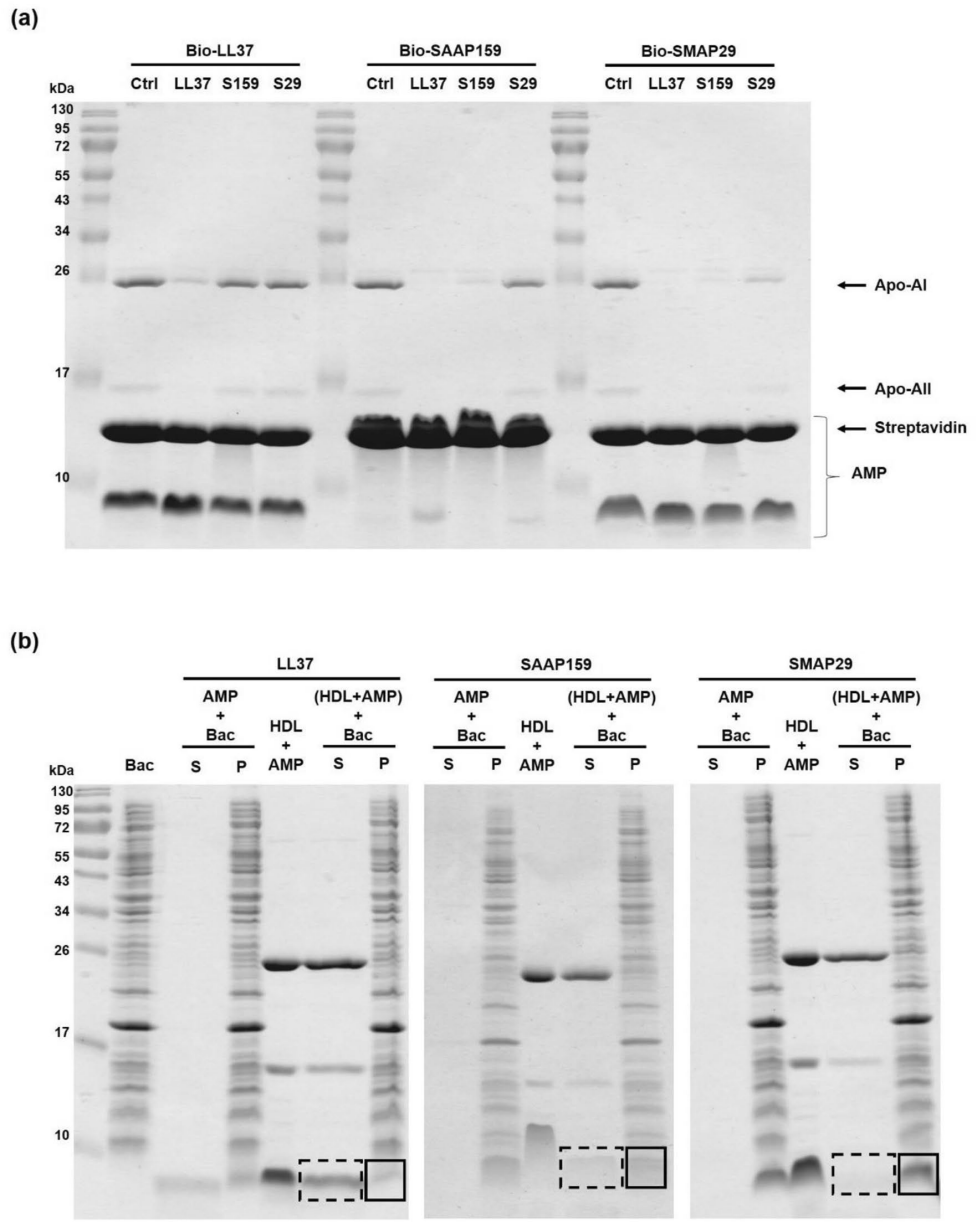
Translocation of AMP from HDL-AMP complex to bacteria. (**a**) Dynamic binding between HDL and AMP. HDL was pulled down by immobilized AMPs, then incubated with twofold amount of free AMP for 30 min at 25 °C. The residual proteins remained on the beads were analyzed by 15% non-reducing SDS-PAGE. (**b**) Translocation of AMP to bacteria. The AMP (2.5 µg) alone or in complex with HDL (5 µg) was incubated with *E. coli* (10^8^ cfu) for 5 min at 25 °C before centrifugation. The proteins in the supernatant (S) and pellet (P) were analyzed by 15% non-reducing SDS-PAGE and Coomassie blue staining. The AMPs translocated to bacteria or remained in soluble complex were shown in solid and dotted lines, respectively.

The HDL components, such as Apo-AI and Apo-AII, immobilized on biotinylated LL37 were significantly competed off by two-fold amounts of free LL37. Interestingly, Apo-AI and Apo-AII immobilized on biotinylated SAAP159 were competed off by free LL37 and SAAP159, but not by SMAP29. Furthermore, those proteins immobilized on biotinylated SMAP29 were nearly competed off by LL37, SAAP159, and only partially by SMAP29 (Fig. 6a). These results indicate that LL37 possesses the strongest binding ability to HDL, then SAAP159 and SMAP29, and the dynamic binding between AMPs and HDL is reversible. The differential binding affinity of HDL to biotinylated AMPs were determined by biolayer interferometry with the following apparent *K*_*D*_ values, 0.11 nM for LL37, 0.40 nM for SAAP159 and 0.36 nM for SMAP29 (Supplementary Table S3, Supplementary Fig. S5).

We subsequently investigated the fate of AMP when the AMP-HDL complexes contact with bacteria by using lipopolysaccharides (LPS), an abundant component of the outer membrane of Gram-negative bacteria, such as *E. coli*, as the surrogate. The LL37 remained in complex with HDL in the supernatant and barely translocated to *E. coli.* in the pellet in the time frame of experiment (Fig. 6b). In contrast, most of the SMAP29 dissociated from the complex and translocated to *E. coli.* In the case of SAAP159, we observed partial translocation of SAAP159 from the complex to bacteria. To assist with interpreting the results of AMP translocations, we further measured the apparent *K*_*D*_ of each AMP in binding to LPS (Supplementary Table S3, Supplementary Fig. S5). Our results indicate that the differential binding affinity of each AMP to HDL and LPS (Supplementary Table S3) appears to govern the translocation behaviors of AMPs (see “Discussion”).

## Discussion

Antimicrobial peptides (AMPs) with the ability to disrupt bacterial membrane have been isolated from multiple sources and some of them are currently in phase II/III clinical trials for the prevention and/or treatment of microbial infections, especially for antibiotic-resistant bacteria^5–7^. However, they are limited to topical infections because they are inhibited in serum. LL37 is found to be bound by human apolipoprotein A-I (Apo-AI) in a 1:1 complex and inhibited its bactericidal and cytotoxic activities^13–15^. Here we have found that the inhibitory effect of serum on AMPs is mediated though HDL instead of apolipoprotein A-I alone, which accounts for approximately 70% of total HDL protein. More importantly, the phospholipids, such as phosphatidylcholine, of HDL played a key role in the binding and inhibition of AMPs. The existence of inhibitory effect of apolipoprotein A-I on LL37 activity reported by Wang et al. may derive from the inclusion of phospholipids in the Apo-AI proteins, which are co-eluted from LL37 affinity column by acids and HPLC C_18_ column by acetonitrile rather than by stepwise elution^13^. In this report, we also purified the Apo-AI by LL37 affinity column, but eluted the Apo-AI and lipids separately by stepwise elution with 30% and 70% TFE, and further analyzed the eluted components by SDS-PAGE and lipid quantitation (Fig. 3a,b).

Thousands of AMPs have been isolated from natural sources. However, some AMPs are susceptible to human serum, while some are not as shown in Fig. 1 and Supplementary Table S1. Here we have found that hydrophobic residues as well as the hydrophobicity of an AMP is critical for its binding to HDL (Fig. 4 and Supplementary Fig. S4), while ionic interactions do not appear to be essential (Supplementary Fig. S2). Most AMPs are known to possess amphipathic structure, cationic and hydrophobic. However, their susceptibilities to the inhibition by serum varied. The helical wheels of AMPs (LL37, TP4, SAAP159, H1a, SMAP29, NRC12 and Pleurocidin) were predicted as shown in Supplementary Fig. S6. All of them exhibited amphipathic structure, but the distribution of hydrophobic residues differed in the hydrophobic face. For example, the hydrophobic residues (Val, Ile, Leu, Phe) of susceptible AMPs, such as LL37, TP4 and SAAP159, are closely clustered, while that (Val, Ile, Leu, Phe, Trp and Tyr) of resistant AMPs, such as NRC12 and Pleurocidin, are intercalated by Ala, Gly and His residues.

HDL play a role in the transportation of lipophilic compounds, such as drugs, and capture of cholesterol and phospholipids from peripheral tissues and release in liver, called reverse cholesterol transport^16–18^. HDL particles (~ 0.6 mg/ml in human plasma) consist of 50% of apolipoproteins (Apo-AI in larger quantity), 20% of free cholesterol (FC) and esterified cholesterol (CE), 15% of phospholipids (PL) and 5% of triglycerides (TG). They are heterogeneous in protein composition, lipid composition, particle size, shape and charge. Several models of HDL structure either in disc or sphere have been proposed with different Apo-AI molecules per particle, such as two in double belt model and three in trefoil model^19^. The core neutral lipids (cholesteryl esters and triacylglycerols) and surface phospholipids (negatively charged phosphatidylcholine) can impact surface fluidity and functions such as cholesterol efflux. The Apo-AI (243 amino acid residues) contains eight amphipathic α-helical domains of 22 amino acids and two repeats of 11 amino acids^20^. Each amphipathic domain is characteristic with hydrophobic and polar faces. Due to the heterogeneity of HDL in composition, the fine structure of native HDL has not been clearly determined by biophysical methods, such as NMR and X-ray crystallography. In this report, we demonstrate that AMP binds to HDL directly through lipids, such as phosphatidylcholine, but not by Apo-AI (Figs. 2e, 3a,b,d).

**Figure 7:**
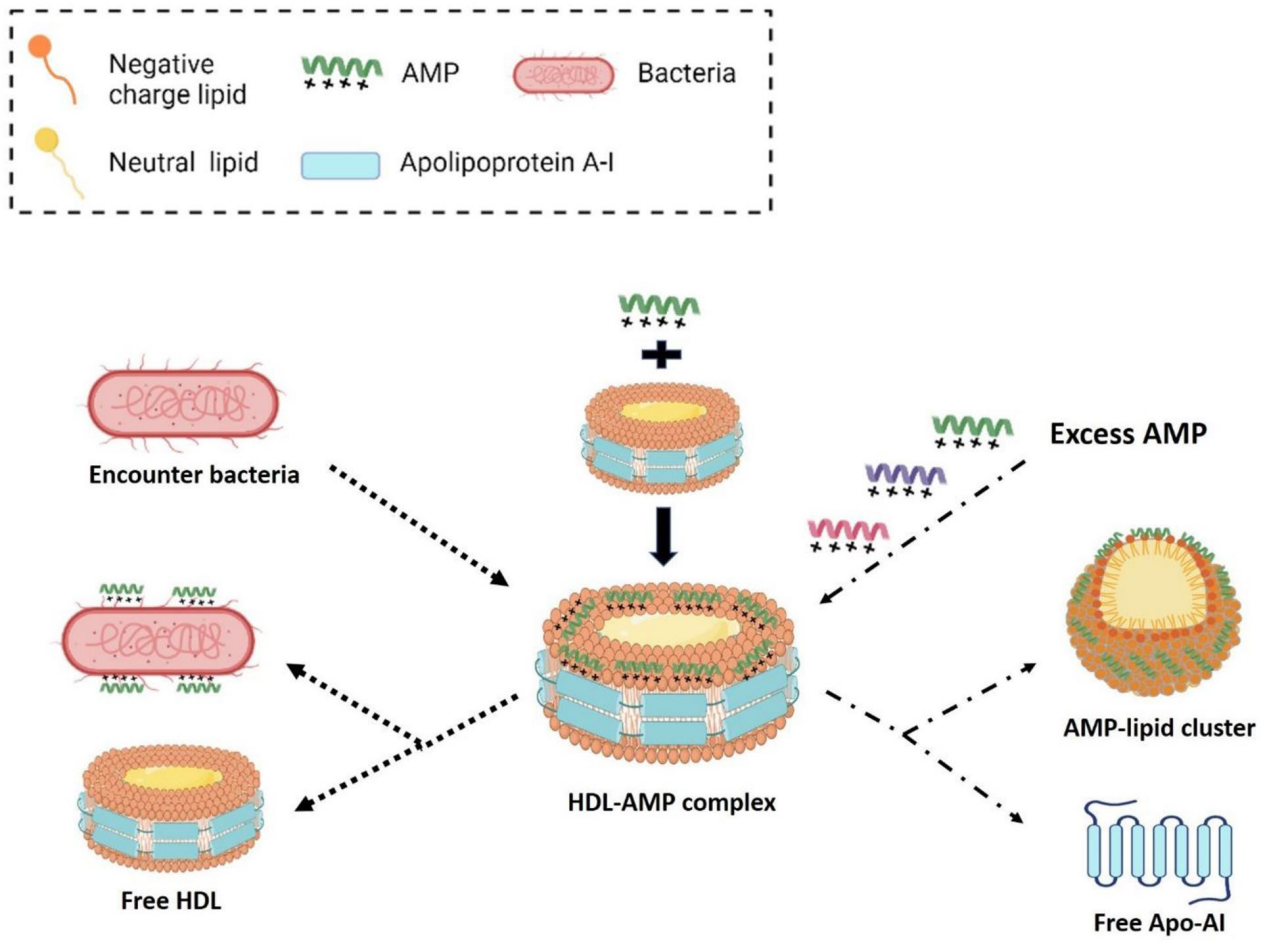
Model of interaction of HDL with AMP and bacteria. Neutral and negatively-charged lipids are assembled in a ring structure along with 8 amphipathic domains of Apo-AI through hydrophobic interaction. Cationic amphipathic AMPs are attached onto lipids mainly by hydrophobic interaction with various affinities. If excess amounts of AMP are embedded in the lipid clusters, the interaction between Apo-AI and lipid cluster of HDL may be weakened leading to the rupture or release of Apo-AI. In the presence of bacteria, the weaker-bound AMPs will dissociate from the AMP-HDL complex and attack to bacteria.

Therefore, it is suggested that neutral and negatively-charged lipids are assembled in a ring structure along with eight amphipathic domains of Apo-AI through hydrophobic interactions. The cationic amphipathic AMPs are then attached onto the negatively-charged lipids by both hydrophobic interactions and ionic strength as proposed in Fig. 7. If excess amounts of AMPs with strong hydrophobicity, such as LL37, are embedded in the lipid clusters, the interactions between Apo-AI and lipid cluster of HDL may be weakened, leading to the rupture or release of Apo-AI. In contrast, if the amount of AMP is not sufficient or the hydrophobicity of AMP is not strong enough, the Apo-AI would remain associated to the AMP-HDL complex.

In the presence of bacteria (LPS), AMPs appear to dissociate from the AMP-HDL complex and translocate to bacteria with different rates. The translocation apparently underlies the bactericidal activities of AMPs. Within the time-frame of experiment, SMAP29 translocated effectively, SAAP159 accomplishes halfway, while LL37 remains mostly in the complex. This behavior appears to be consistent with the differential binding affinity of each AMP to HDL and LPS (Supplementary Table S3). The higher the HDL to LPS apparent *K*_*D*_ ratio, the more efficient is the translocation. We do not know whether other factors, such as salts and divalent cations, also contribute to the AMP translocation. If apparent *K*_*D*_*s* alone are indeed the deciding factors for the efficiency of translocation, potential AMPs with higher HDL/LPS *K*_*D*_ ratio, like NRC12, can be selected or engineered for the applications in systematic therapy of bacterial infection.

In conclusion, AMPs are bound by HDL in human blood. The binding is mainly mediated through hydrophobic interactions between the phospholipids of HDL and the hydrophobic regions of AMP. The AMP-HDL complex may server as a carrier of AMP for attacking bacteria upon encountering microbial infection in the human body.

## Methods

### Materials

Antimicrobial peptides LL37^21^, TP4^22^, SMAP29^23^, CAME^24^, RR12^24^, RRIKA^24^, SAAP159^7^, GW-Q6^25^, H1a^25^, Pilosulin-1^26^, BMAP27^27^, Lartarcin-2a^28^, NRC12^29^, Pleurocidin^30^ and their biotinylated derivatives, were synthesized by Kelowna International Scientific Inc. (Taipei, Taiwan) with more than 95% purity and their molecular masses were verified by mass spectrum analysis. Polymyxin B (PXB), 8-anilino-1-naphthalenesulfonic acid (ANS), lipopolysaccharides (LPS), sphingomyelin, cholesteryl oleate and phosphatidylcholine were purchased from Sigma-Aldrich (St. Louis, Missouri, USA). The mouse antibody raised against apolipoprotein AI (GTX 15659) was obtained from Genetex (San Antonia, USA). The rabbit antibodies raised against LL37 and TP4 were prepared by Yao-Hong Biotechnology (New Taipei, Taiwan) using synthetic polypeptides as immunogens. Human serum samples were collected and prepared from adult human in accordance with the guidelines of Academia Sinica IRB. The informed consent was obtained from all blood donors. The study protocol was approved by Academia Sinica, Taiwan, ethics committee with the approval number AS-IRB01-18072. The informed consent was obtained from all blood donors. Streptavidin-conjugated beads were obtained from GE Healthcare Bio-sciences AB (Uppsala, Sweden). *E. coli* K-12 (MG1655) was purchased from Food Industry Research and Development Institute (Hsinchu, Taiwan). 2,2,2-trifluoroethanol (TFE) was purchased from Acros Organics (New Jersey, USA). Sodium N-dodecanoylsarcosinate (sarkosyl) was supplied by Wako Pure Chem. (Osaka, Japan).

### Antimicrobial activity assays

The Gram-negative bacteria *Escherichia coli K-12* (MG1655) was cultured and plated in/on Luria–Bertani broth/agar plate (Merck Millipore, Darmstadt, Germany). The bacteria were grown overnight, washed, and diluted 1:2000 in phosphate buffered saline (PBS), pH 7.4. Bacteria (ca. 1 × 10^6^ colony-forming units, cfu/ml) was mixed with antimicrobial peptides (AMP) in 100 µl PBS and incubated at 37 °C. Serial dilutions of each AMP-treated bacteria in the presence of human sera, proteins or lipids were prepared and plated for determination of remaining viable cells (expressed as cfu). At least three independent experiments were performed for each assay to determine the mean value with standard deviation (SD)^31^. LC_99_ was defined as the lowest AMP concentration that kills 99% of the original inoculum.

### Band shift of AMPs or HDL on native PAGE

Various AMPs (4 µg each) were incubated with human serum, isolated components or lipid at the indicated concentrations at room temperature in 10 µl PC buffer (20 mM HEPES, pH7.4, 50 mM NaCl) for 30 min, subjected to horizontal native 8% polyacrylamide gel electrophoreses (PAGE), pH8.0/pH8.8, and stained with Coomassie Blue.

### Identification and purification of AMP-binding proteins from human serum

Ten µg of biotinylated LL37 was incubated with 10 µl of streptavidin-conjugated beads in 600 µl PC buffer for one hour on a rolling wheel. The crude serum was incubated with immobilized AMPs at room temperature for 30 min on a rolling wheel, then washed twice with the respective buffer and subjected to SDS-PAGE and Coomassie Blue staining. Protein bands were excised from the SDS-PAGE and subjected to in-gel trypsin digestion and liquid chromatography-tandem mass spectrometry (LC/MS/MS)^32^. The proteins/lipids bound to the immobilized AMPs were stepwise eluted with 30%, 50% or 70% TFE, vacuum dried and dissolved in PC buffer containing 2% TFE.

#### Analysis of AMP translocation by SDS-PAGE

LL37, SAAP159 and SMAP29 (4 µg) alone or in complex with HDL were incubated with *E. coli* (10^8^ cfu) in 50 µl of PC buffer for 5 min at room temperature and subjected to centrifugation at 800 g for 5 min. The AMP and HDL in the supernatant as well as that bound to bacteria in the pellet were dissolved in SDS-loading buffer and subjected to SDS-PAGE analysis and Coomassie Blue staining.

#### Morphology of S50, Apo-AI and HDL analyzed by transmission electron microscopy (TEM)

Samples (0.5 µg of Apo-AI, HDL and S50) were dissolved in 20 µl of PC buffer, then loaded onto copper grid (300 mesh) using a filter paper to blot off excess solution. The grids were washed three times with ddH_2_O, stained with 1% uranyl acetate (EMS, Pennsylvania, USA) and examined under a transmission electron microscope Tecnai G2 Spirit TWIN (Thermo)^33^.

#### Measurement of ANS fluorescence

Small volumes of ANS stock were stepwise added to a 200 µl 10% TFE solution containing 4 µg of AMP at the final concentrations of 10, 20, 30, 40 and 50 µM. The emission spectra of ANS excited at 380 nm were measured between 400 and 600 nm using a temperature-controlled spectrofluorometer (FP-8500 Jasco, Japan). The free form ANS exhibits an emission maximum at 520 nm while the peptide-bound ANS exhibits an emission maximum mainly at 470 nm^34^.

### Lipid quantification

The lipids of TFE eluates (E1–E6 and S50), Apo-AI and HDL were determined by a Lipid Quantification Kit (Colorimetric, ab65390) from Abcam (Cambridge, MA) in accordance with the manufacturer’s instructions. Briefly, first, samples were incubated with 18 M sulfuric acid at 90 °C for 10 min, then mixed with vanillin reagent and further incubated at 37 °C for 15 min. The absorbance of unsaturated fatty acids (UFAs) was measured at the wavelength of 540 nm. The concentrations of lipids were determined using the manufacturer-provided lipids as a standard.

### Binding kinetics of HDL and LPS to biotinylated antimicrobial peptides

Biotinylated AMPs (10 µM) were immobilized on a streptavidin biosensor which was pre-incubated with 10 mg/ml of bovine serum albumin (BSA) for 30 min. Serial dilutions of HDL and lipopolysaccharide (LPS) in PC buffer were used as analyte. The biolayer interferometry was measured using an Octet RED96e instrument (ForteBio). Affinity (*K*_*D*_) and kinetic parameters (*k*_*on*_ and *k*_*off*_) were calculated from a global fit (1:1) of the data using the Octet RED96e software^35^.

### Statistical analysis

For all in vitro antimicrobial assays, the average value with standard deviation (SD) was determined from at least three independent experiments. Mann–Whitney test and Kruskal–Wallis tests were performed to determine the significance. For lipid quantification, the average value with standard deviation (SD) was determined from at least three independent experiments and One-way ANOVA test was performed to determine the significance of the difference in the measured values. *P* < 0.05 was considered to be statistically significant.

## Supplementary Information

Supplementary Information.

## Data Availability

The data that support the findings of this study are available from the corresponding author upon reasonable request.
