# Supplementary material for: The lipid components of high-density lipoproteins (HDL) are essential for the binding and transportation of antimicrobial peptides in human serum
Source: Sci Rep. 2022 Feb 16;12:2576. doi: 10.1038/s41598-022-06640-7 (PMC8850444; doi:10.1038/s41598-022-06640-7)
Supplement: Supplementary file 1 — Supplementary Information. [file 41598_2022_6640_MOESM1_ESM.pdf]

Supplementary material

The lipid components of high-density lipoproteins (HDL) are essential for the binding and transportation of antimicrobial peptides in human serum

Wen-Hung Tang[1], Shi-Han Wang[1], Chiu-Feng Wang[1], Yun Mou[1], Min-Guan Lin[2], Chwan-Deng Hsiao[2] and You-Di Liao[1*]

1. Institute of Biomedical Sciences, Academia Sinica, Taipei 115, Taiwan
2. Institute of Molecular Biology, Academia Sinica, Taipei 115, Taiwan

Contents:

Supplementary Tables 1-3

Supplementary Figures 1-18

Supplementary Table 1. Reduced bactericidal activities of antimicrobial peptides against *E. coli* in human serum.

AMPs	LC$_{99}$ (mg/L) against *E. coli*		
	PBS	5% Serum in PBS	Fold increased (X)
Group 1			
LL37	16	>512	>32
TP4	8	128	16
RRIKA	8	128	16
SAAP159	1	16	16
H1a	16	128	8
Pilosulin-1	16	128	8
RR12	4	32	8
Group 2			
BMAP27	1	2	2
GW-Q6	8	16	2
Lartarcin-2a	8	16	2
SMAP29	4	4	1
CAME	8	8	1
NRC12	32	32	1
Pleurocidin	16	16	1

Supplementary Table 2. List of interested proteins identified from human serum by LC/MS/MS.

Protein	Accession # of UniProKB	Protein	Score	# of Identified Peptides	Sequence Coverage (%)	Molecular weight (kDa)
Band I	P02647	Apolipoprotein A-I	8597	40	77	28.3
Band II	V9GYE3	Apolipoprotein A-II	3927	187	55	17.4

The MS raw data were searched against the UniProtKB HUMAN database.

Supplementary Table 3. The binding affinity of biotinylated AMPs to HDL and LPS determined by biolayer interferometry.

Bio-AMP	K_D (nM)	
	HDL	LPS
LL37	0.11	0.33
SAAP159	0.40	0.26
SMAP29	0.36	0.04
NRC12	5.27	0.02

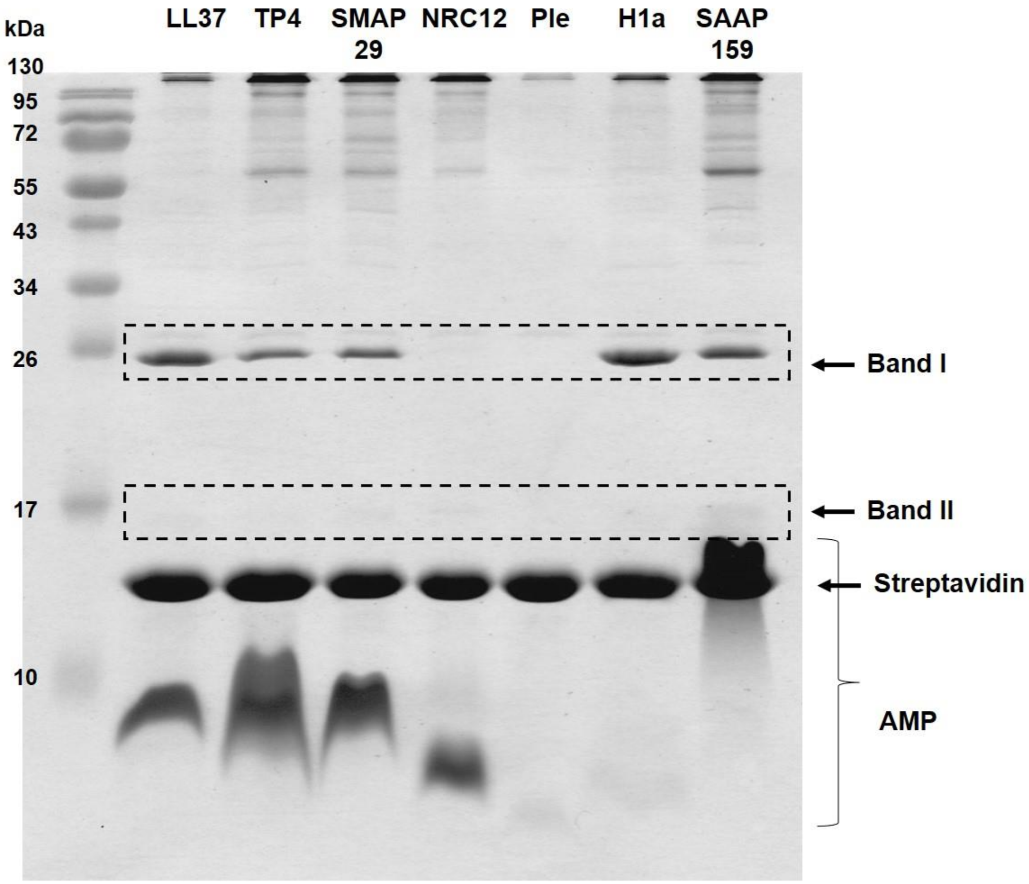

Supplementary Figure 1. Serum proteins were bound to various biotinylated AMPs. The components of crude human serum were pulled down by biotinylated AMPs which have been immobilized on Streptavidin-conjugated beads, and the residual components on the beads were analyzed by 15% non-reducing SDS-PAGE and Coomassie blue staining.

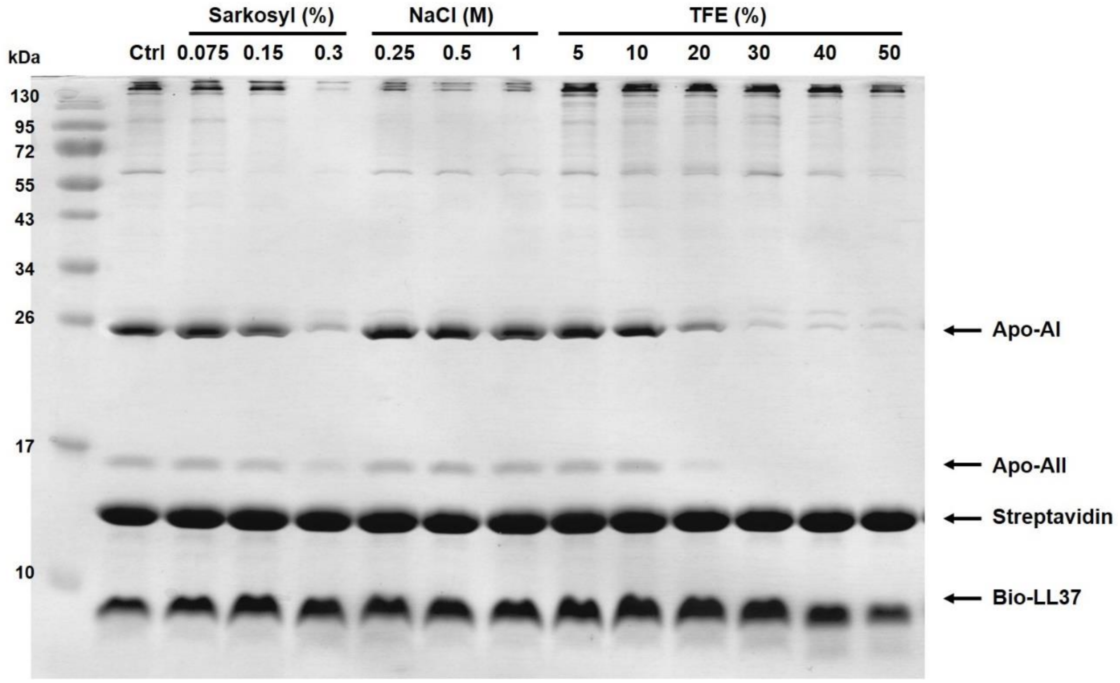

Supplementary Figure 2. AMP-binding stability of serum proteins to various solvents. The serum components were pulled down by biotinylated LL37 which were immobilized on the Streptavidin-conjugated beads, washed by various agents at the indicated concentrations and the residual proteins were analyzed by 15% non-reducing SDS-PAGE and Coomassie blue staining.

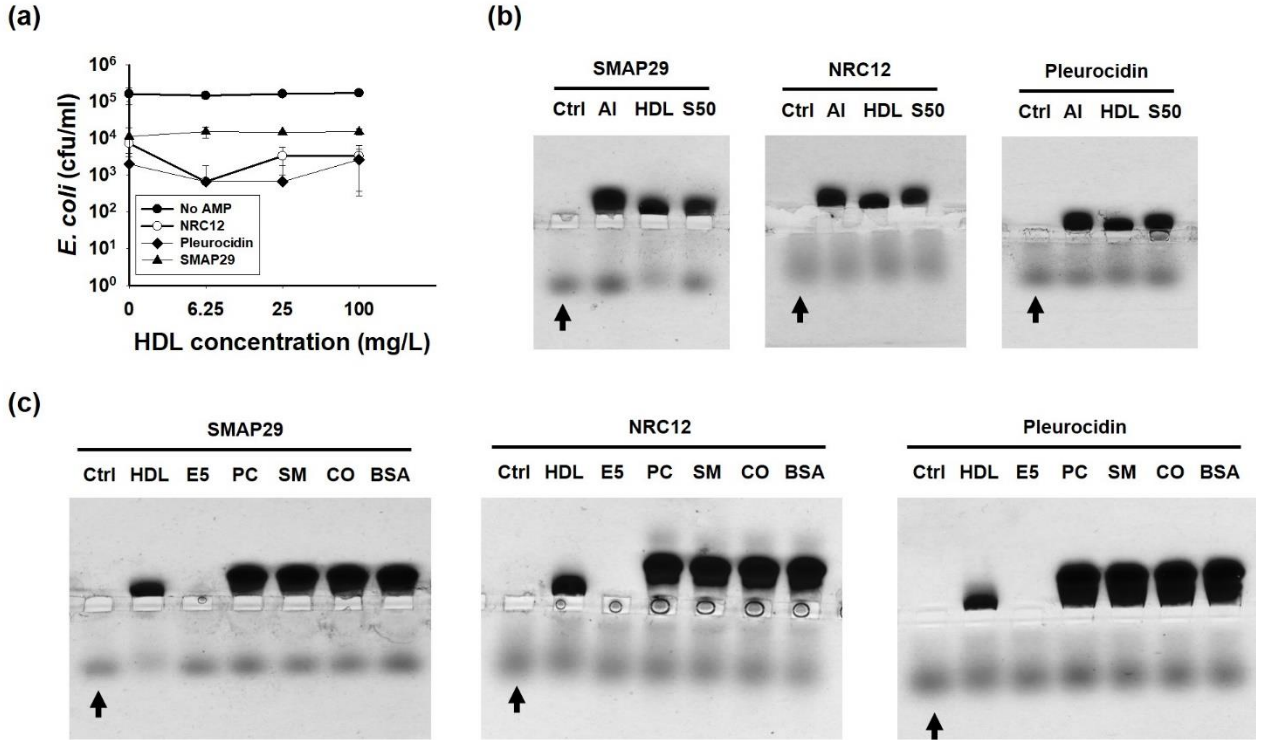

Supplementary Figure 3. Susceptibility of SMAP29, NRC12 and Pleurocidin to the inhibition by Apo-AI, S50 and HDL. (**a**) Effect of HDL on antimicrobial activities of SMAP29 (1mg/L), NRC12 (16mg/L) and Pleurocidin (8mg/L) against *E. coli* (10^6 cfu/ml). Values are the mean ± SD (n=3). Kruskal-Wallis test was performed to determine the significance of the difference in each group. (**b**) The band shift of AMPs (4μg each) by Apo-AI, S50 and HDL (10μg each) analyzed by 8% horizontal native PAGE and Coomassie blue staining. (**c**) Band shift of AMPs by 10μg HDL, E5 and various lipids (4μg each, dissolved in 20μg/10μl BSA) and analyzed by 8% horizontal native PAGE, pH8.0, and Coomassie blue staining. Arrow indicates control (non-shift) AMP. PC: phosphatidylcholine; SM: sphingomyelin; CO: cholesteryl oleate; BSA: bovine serum albumin.

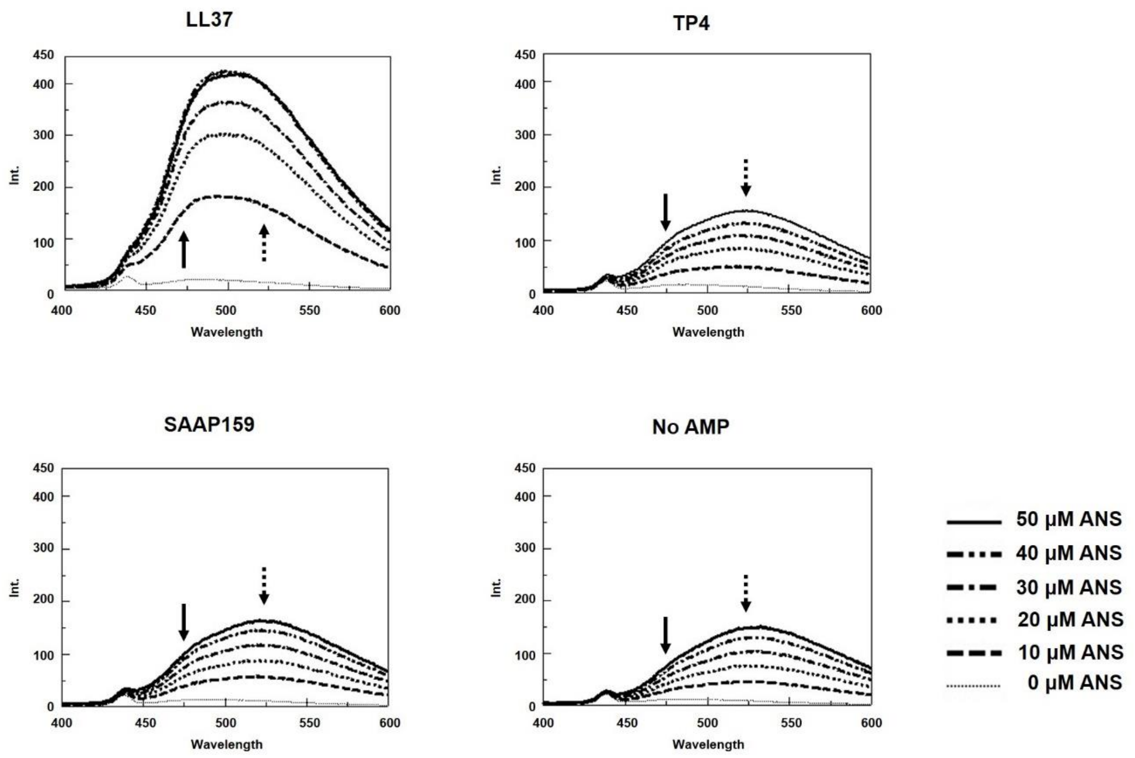

Supplementary Figure 4. Hydrophobicity of AMPs. Emission spectra of 4μg AMPs dissolved in 200μl 10% TFE. ANS was added to the AMP solutions at the indicated final concentrations, excited at 380 nm and recorded for the emission spectrum between 400 to 600 nm. The free- and bound-form ANS exhibited an emission maximum at 520 nm (dotted arrow) and 470 nm (solid arrow), respectively.

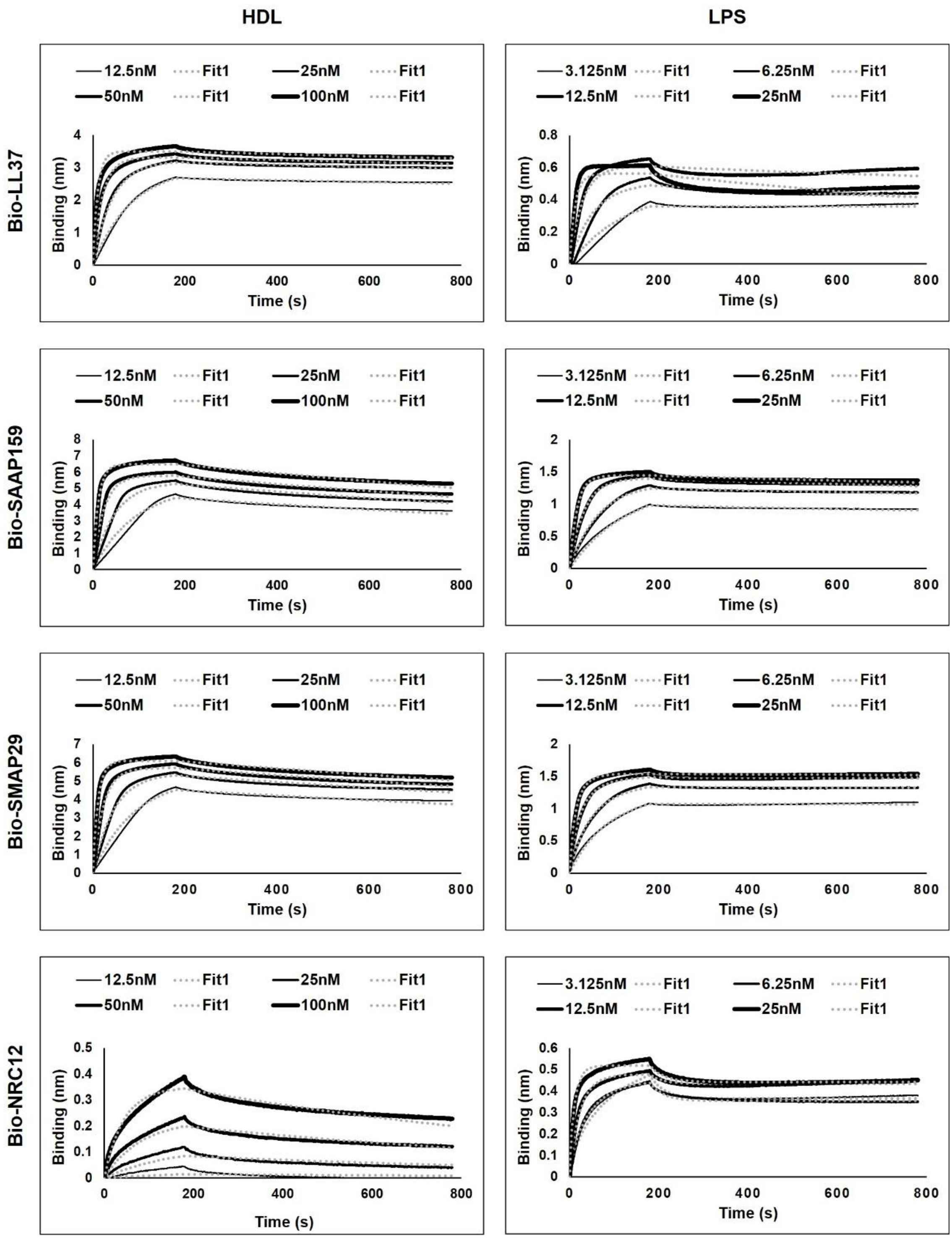

Supplementary Figure 5. Binding kinetics of biotinylated AMPs to HDL and LPS measured by biolayer interferometry. Heavy lines represent the experimental data, dotted lines represent the global-fitted data for analysis.

(a)

1. **LL37:** LLGDFFRKSKEKIGKEFKRIVQRIKDFLRNLVPRTES
2. **TP4 (Piscidins 4):** FIHHIIGGLFSAGKAIHRLIRRRRR
3. **SAAP159:** LKRLYKRVFRLLKRYYRQLRRPVR
4. **H1α:** GYNYAKKLANLAKKFANALW
5. **SMAP29:** RGLRRLGRKIAHGVKKYGPTVLRIIRIAG
6. **NRC12:** GWKKWFNRAKKVGKTVGGLAVDHYL
7. **Pleurocidin:** GWGSFFKKAAHVGKHVGKAALTHYL

(b)

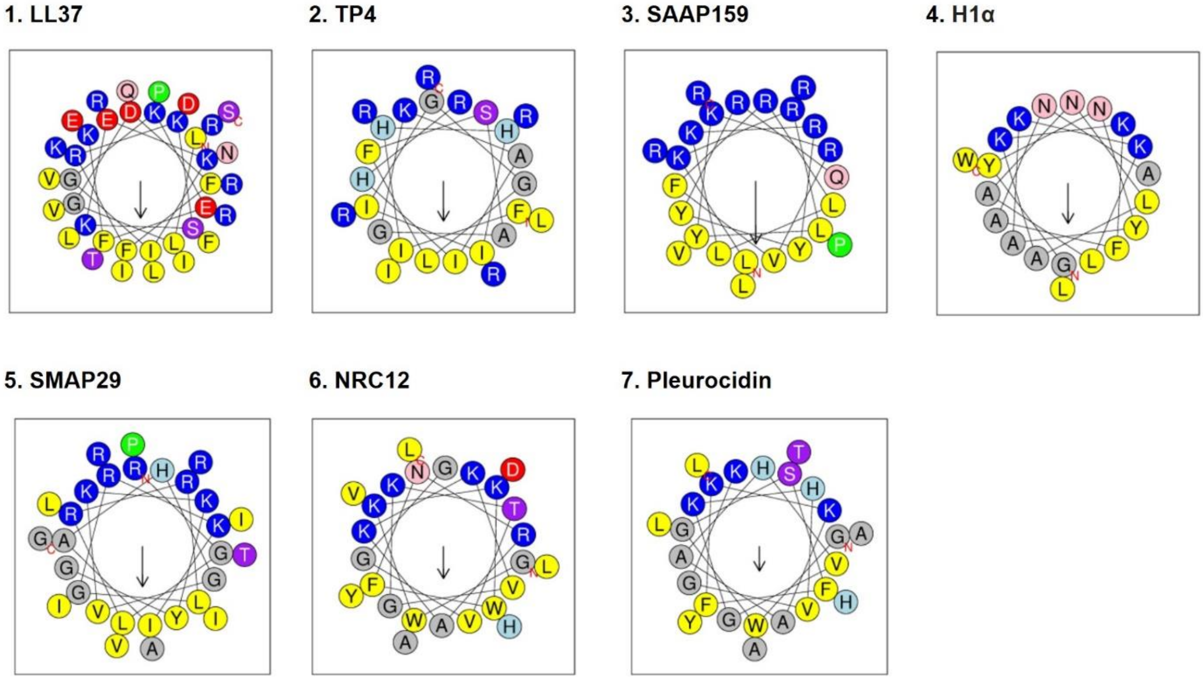

Supplementary Figure 6. Predicted structure of AMPs. (**a**) Amino acid sequence of AMPs. (**b**) The amphipathic α-helical structures of AMPs were simulated by the heliquest program (https://heliquest.ipmc.cnrs.fr/cgi-bin/ComputParams.py). The cationic, anionic and hydrophobic residues are shown in blue, red and yellow, respectively.

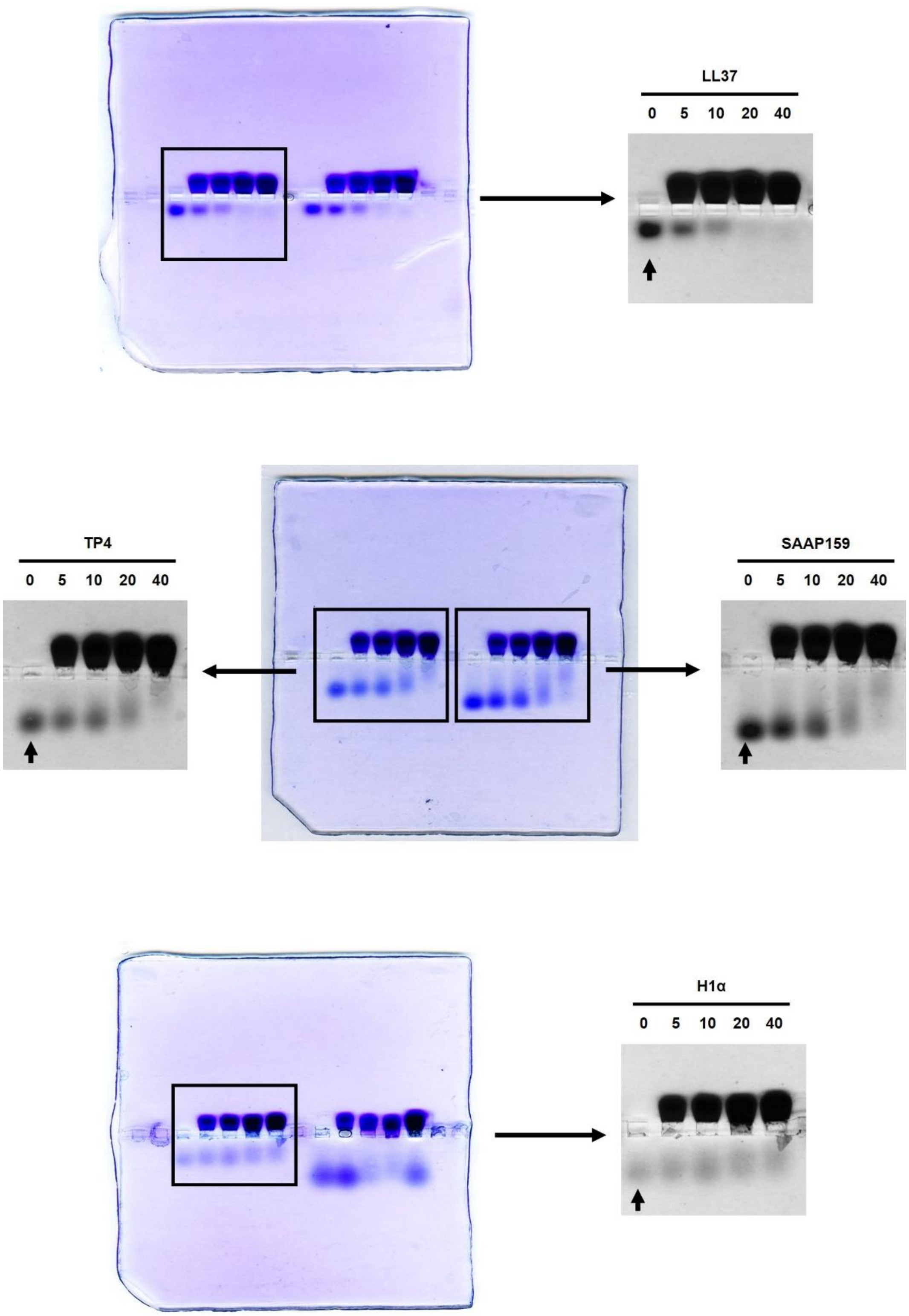

Supplementary Figure 7. Demonstration of original and final gels which were used in Figure 1b top panel.

bottom panel.

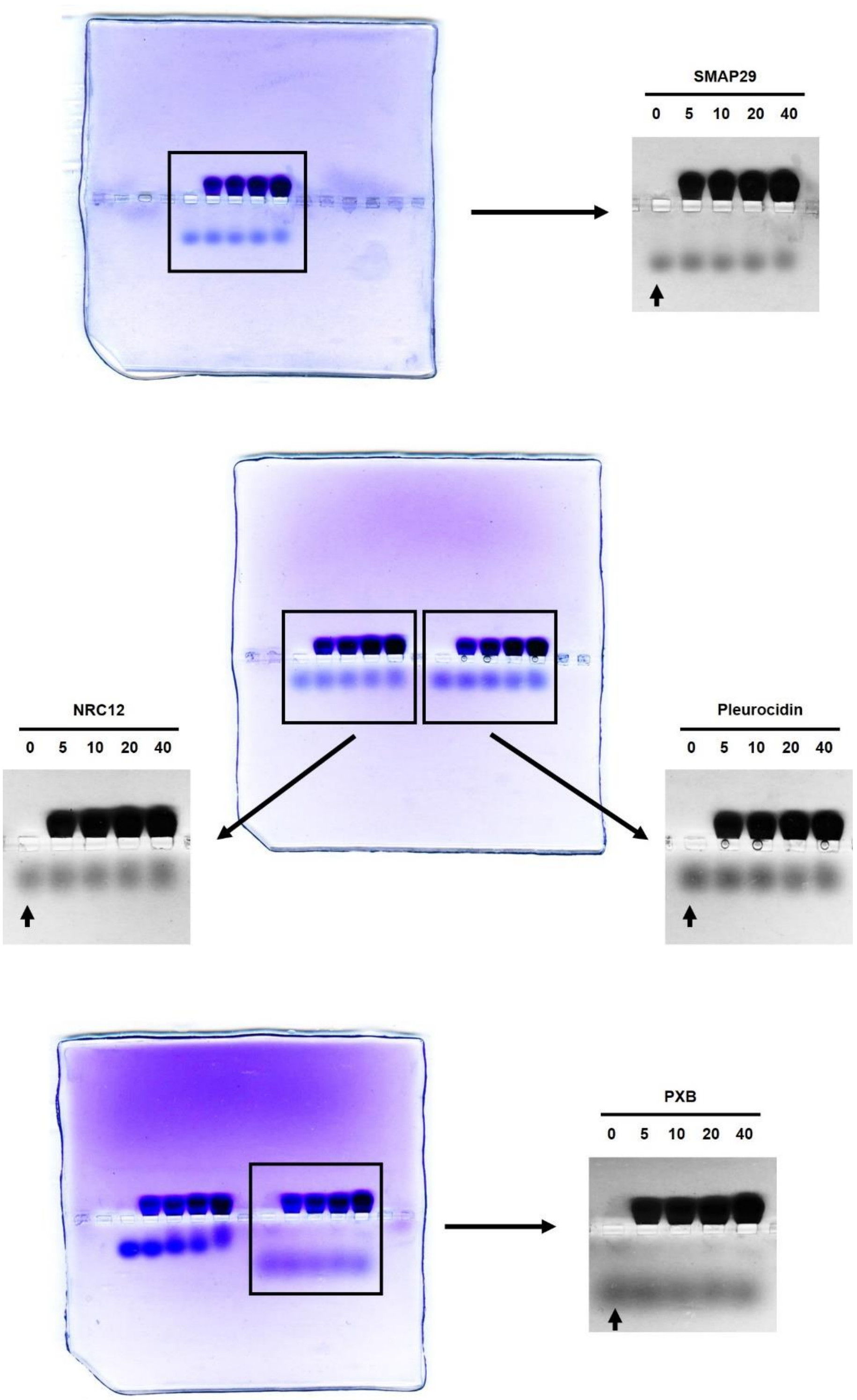

Supplementary Figure 8. Demonstration of original and final gels which were used in Figure 1b bottom panel.

(a)

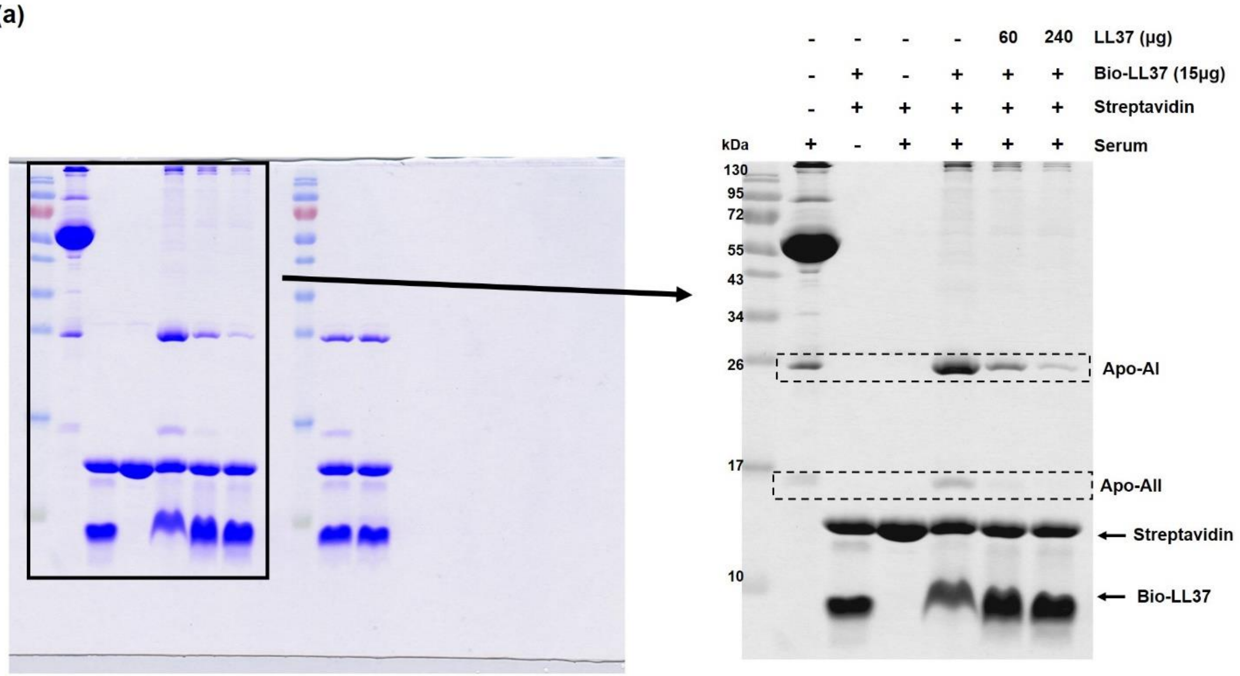

(b)

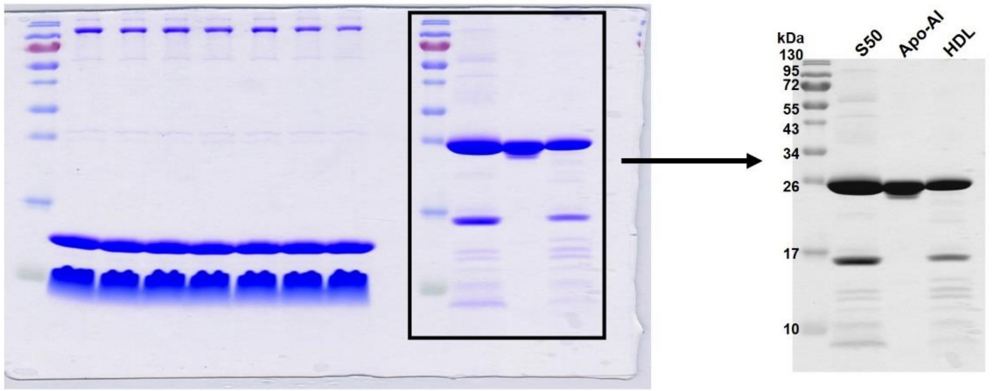

Supplementary Figure 9. Demonstration of original and final gels which were used in Figure 2a (**a**) and Figure 2b (**b)**.

(a)

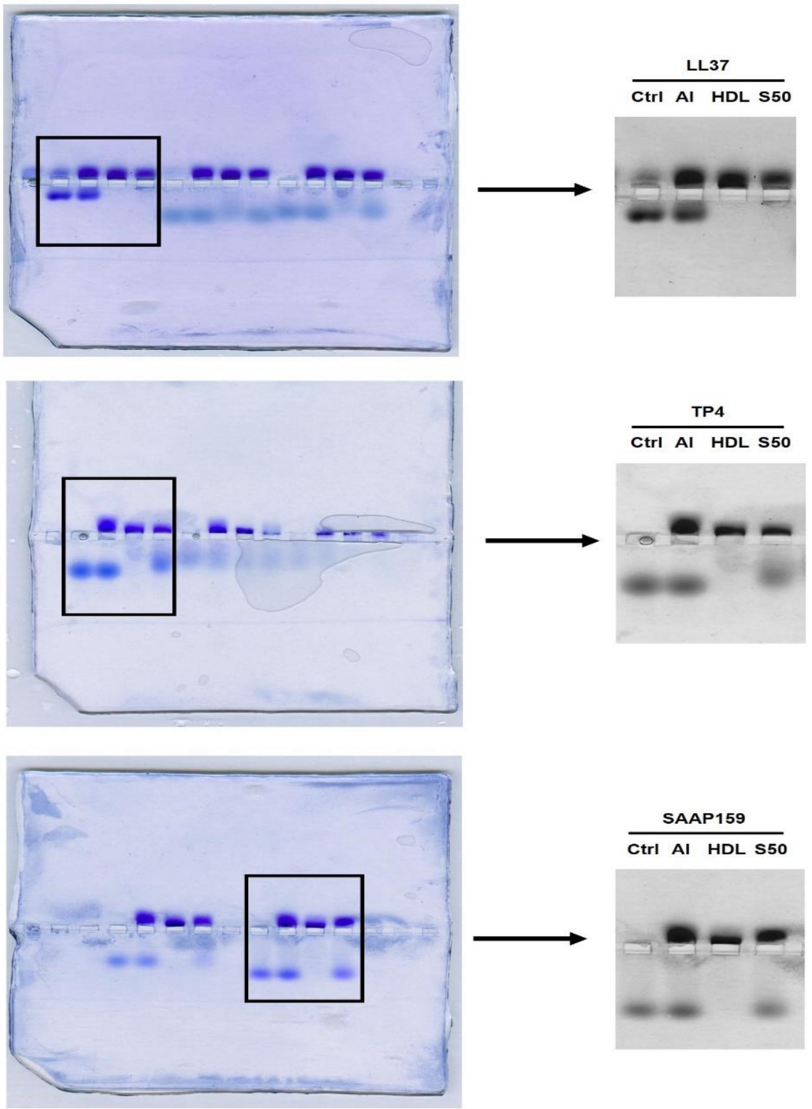

(b)

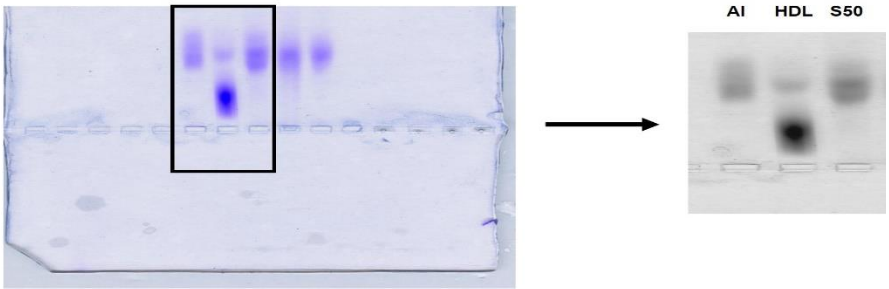

Supplementary Figure 10. Demonstration of original and final gels which were used in Figure 2f (**a**) and Figure 2g (**b**).

(a)

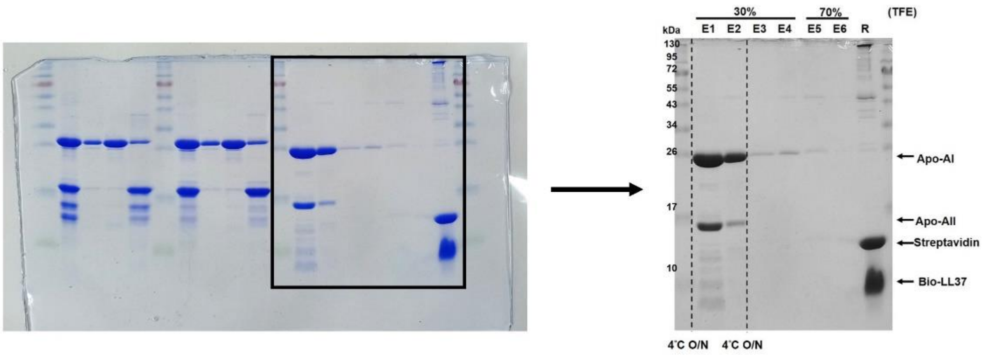

(b)

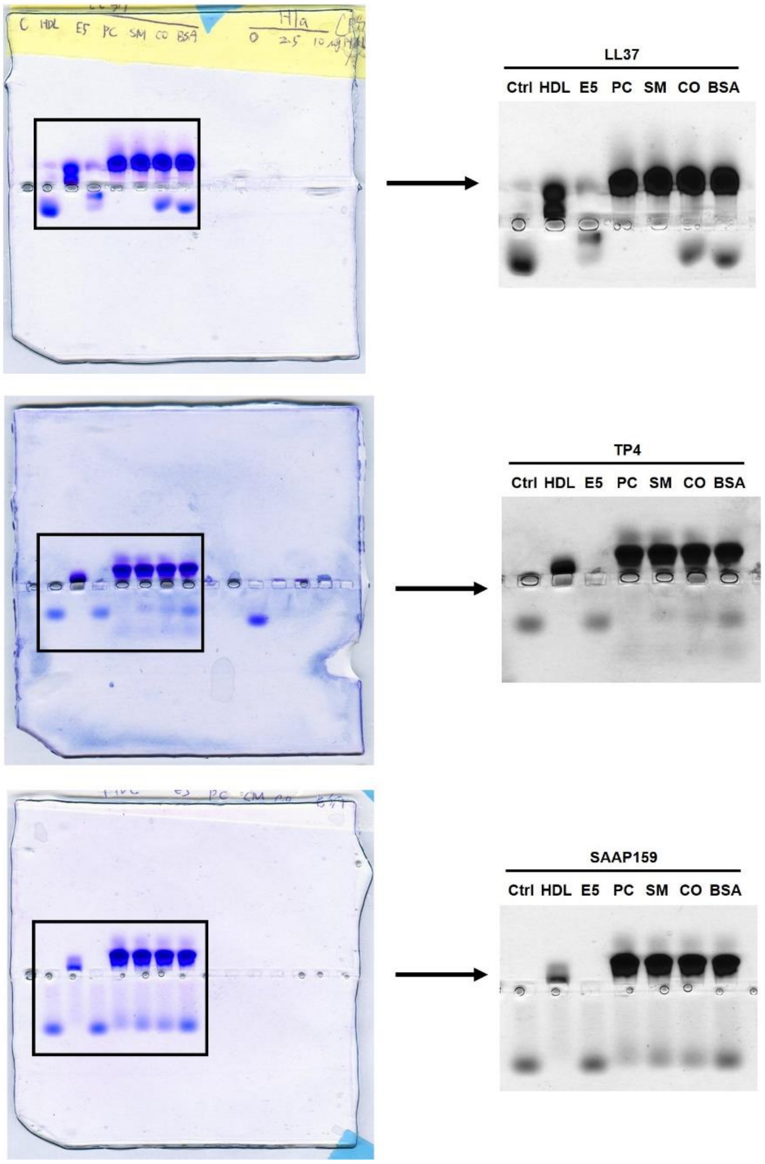

Supplementary Figure 11. Demonstration of original and final gels which were used in Figure 3a (**a**) and Figure 3d (**b**).

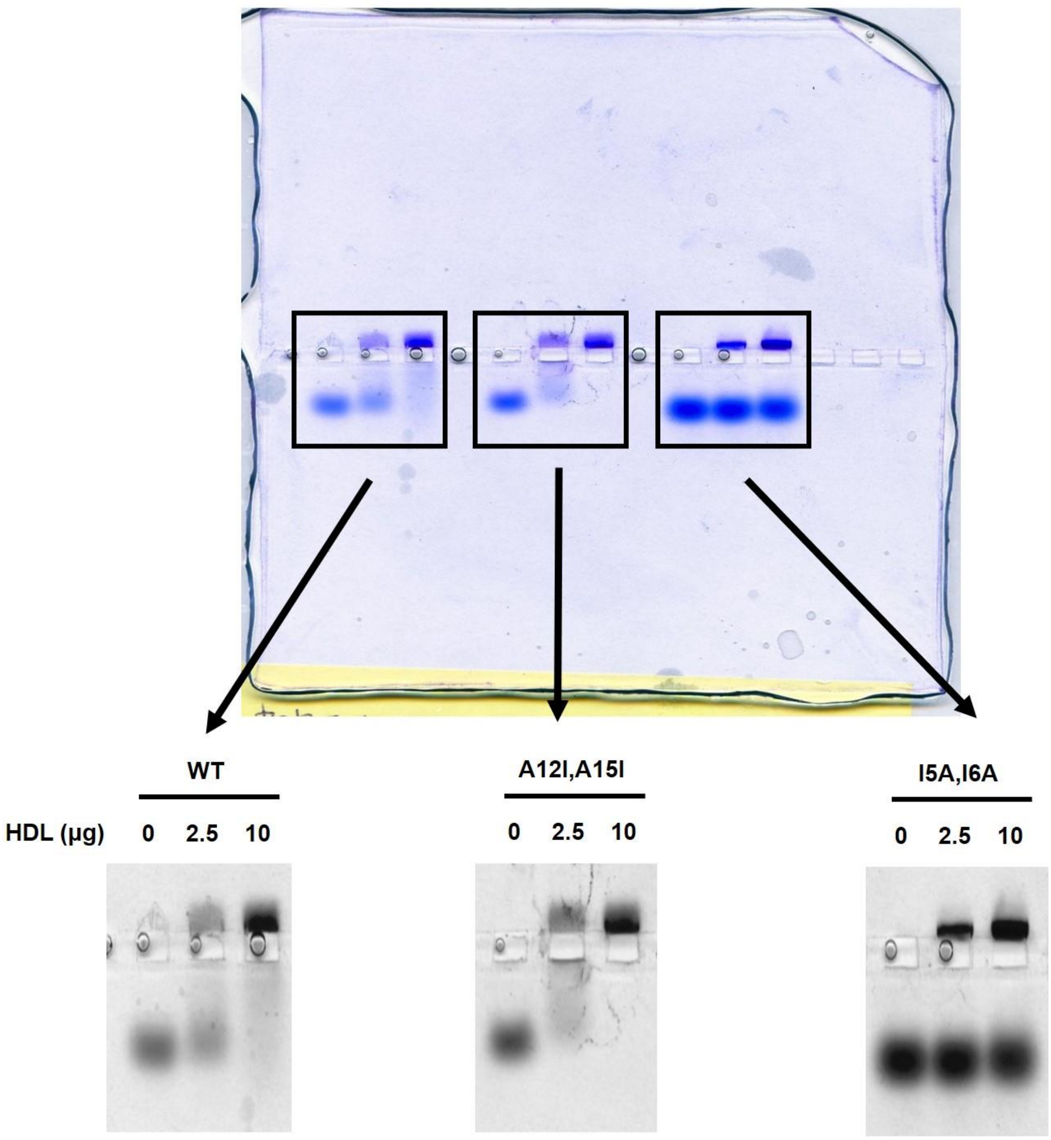

Supplementary Figure 12. Demonstration of original and final gels which were used in Figure 4b.

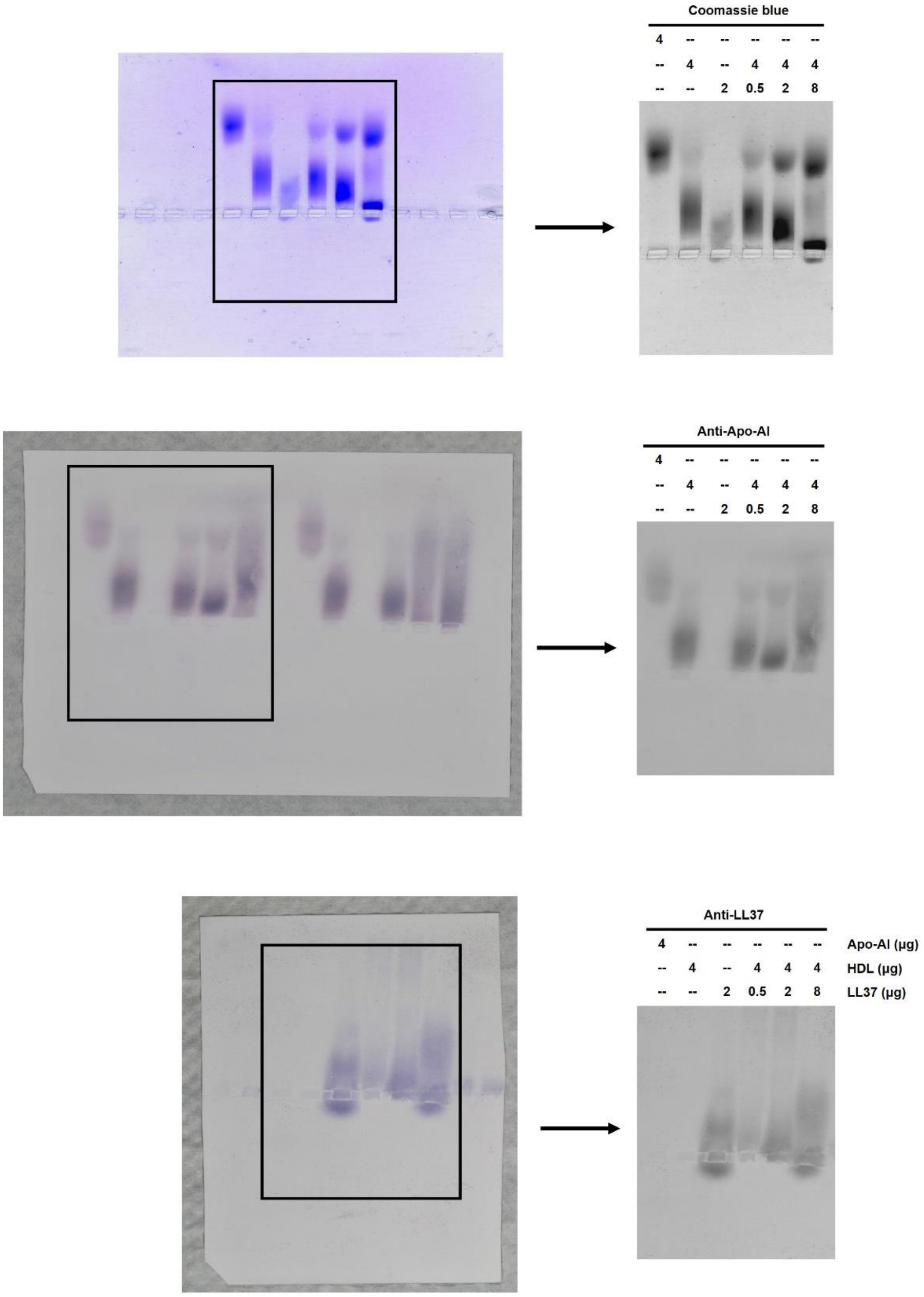

Supplementary Figure 13. Demonstration of original and final gels which were used in Figure 5a.

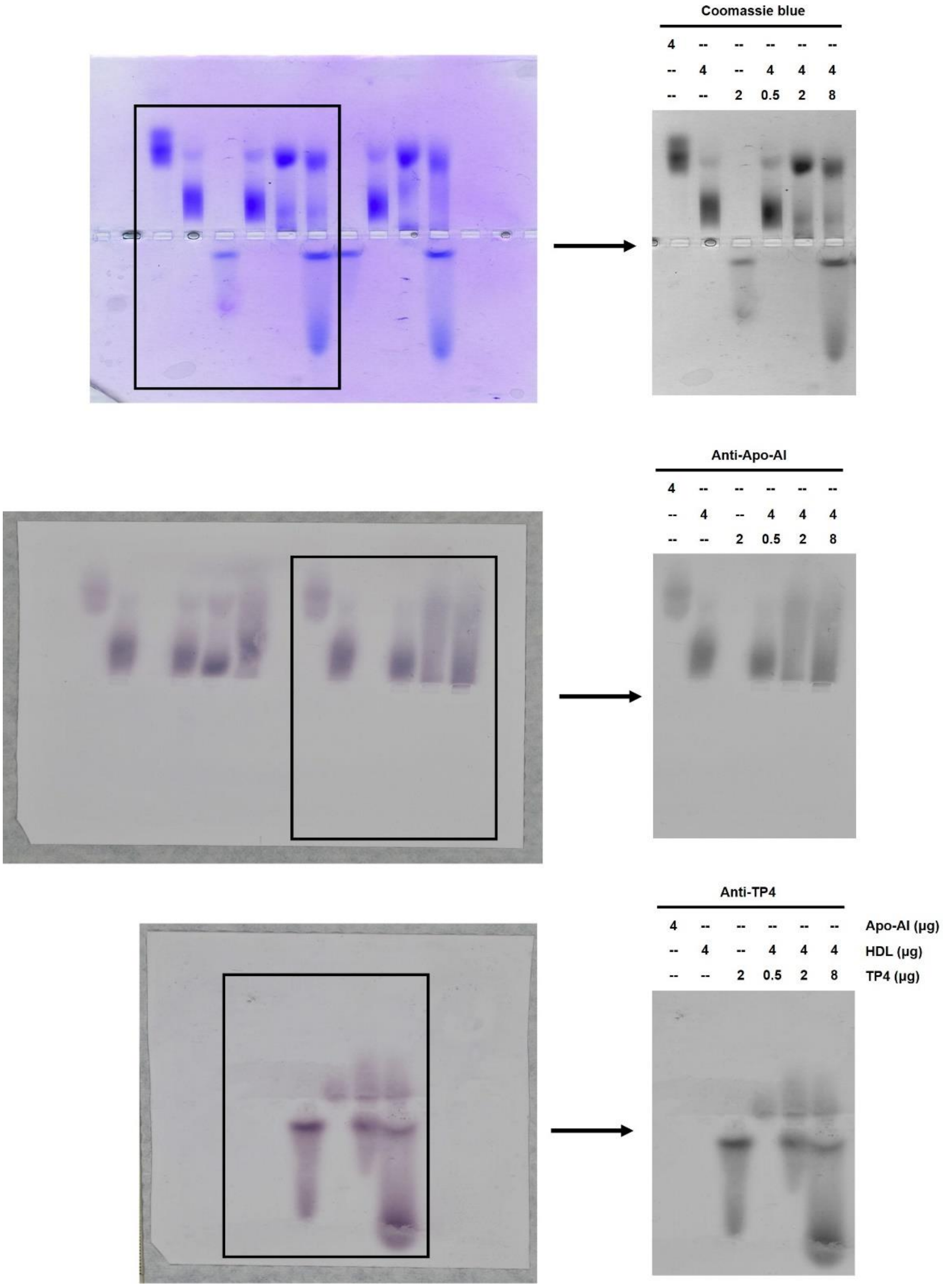

Supplementary Figure 14. Demonstration of original and final gels which were used in Figure 5b.

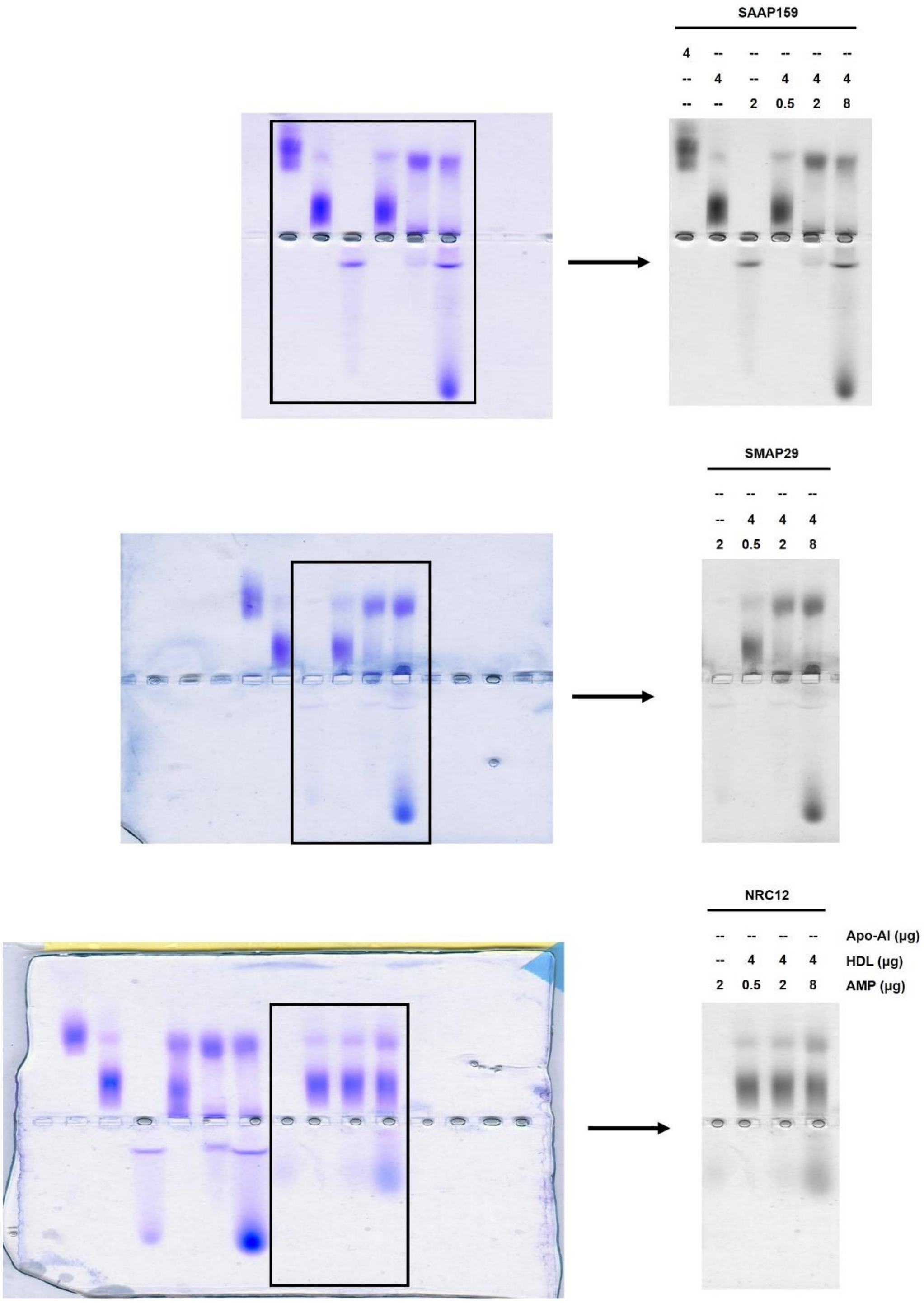

Supplementary Figure 15. Demonstration of original and final gels which were used in Figure 5c.

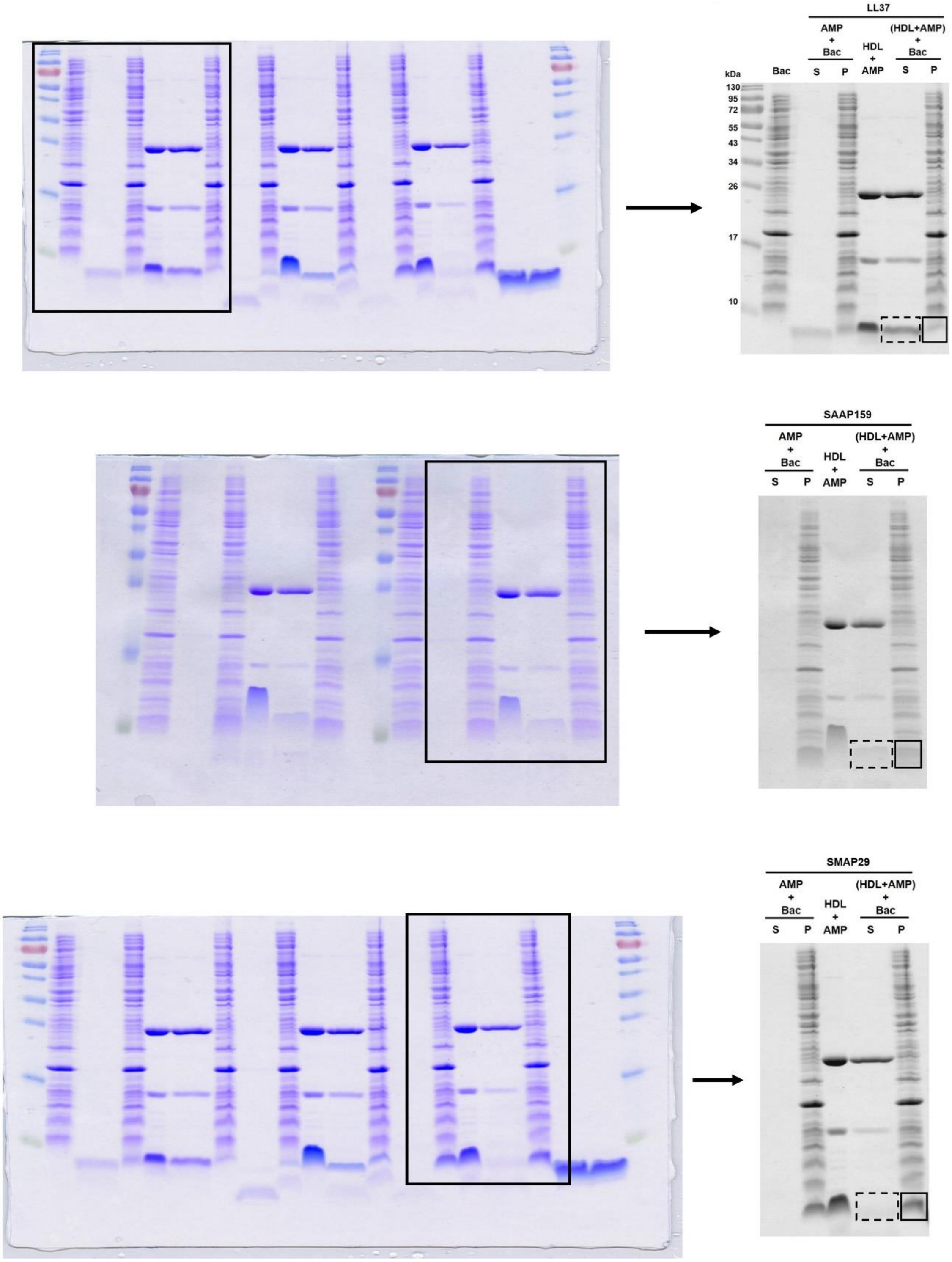

Supplementary Figure 16. Demonstration of original and final gels which were used in Figure 6b.

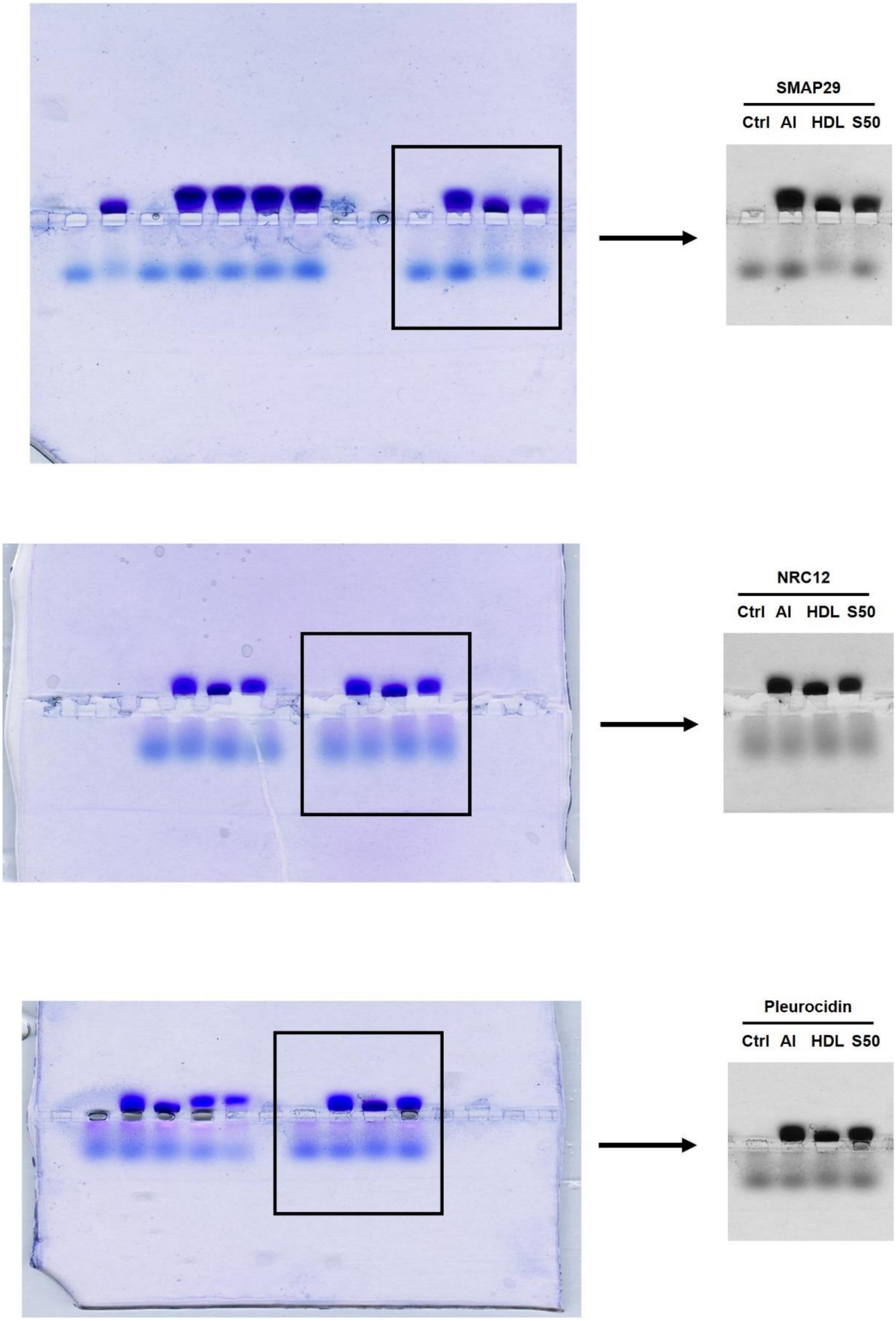

Supplementary Figure 17. Demonstration of original and final gels which were used in Figure S3b.

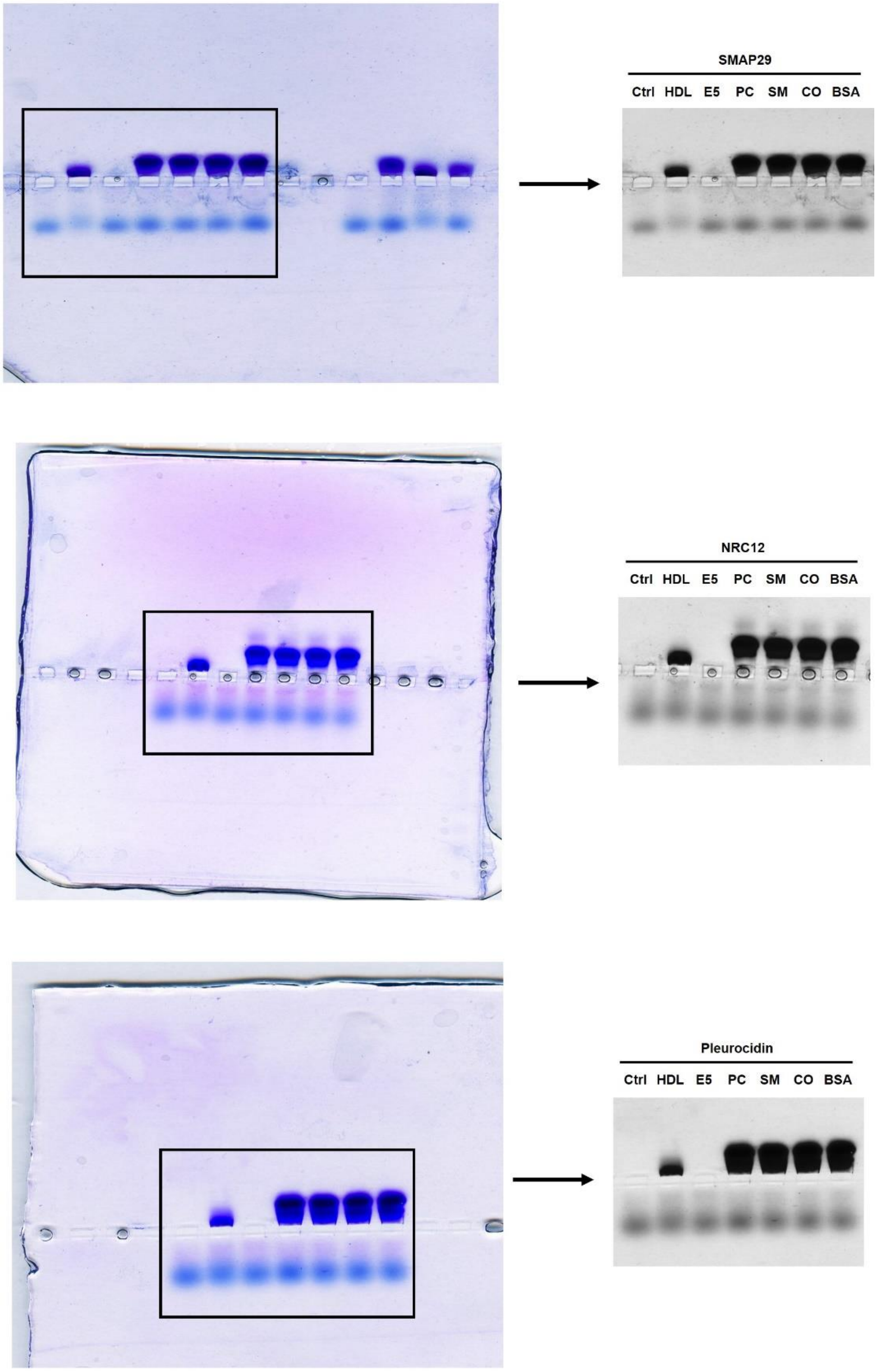

Supplementary Figure 18. Demonstration of original and final gels which were used in Figure S3c.
